# mRNA delivery systems 2.0: Engineering extrahepatic delivery for non-vaccine therapeutics

**DOI:** 10.1016/j.mtbio.2025.102584

**Published:** 2025-11-21

**Authors:** Manoj Dalabehera, Arnab Ghosh, Satyajit Mohanty, Dinesh Kumar Chellappan, Shubham Chaudhari, Yogita Ale, Neelam Poonia, Rudra Narayan Subudhi, Manmeet Kaur Khanna, Hae Gyun Lim

**Affiliations:** a Department of Pharmaceutics, Uttaranchal Institute of Pharmaceutical Sciences, Uttaranchal University, Dehradun, 248007, Uttarakhand, India; b Biomedical Ultrasound Lab, Department of 4th Industrial Convergence Bionics Engineering, Pukyong National University, Busan, South Korea; c Team Photonics & Metabolics, Division of Pharmacology, Department of Pharmaceutical Sciences and Technology, Birla Institute of Technology, Mesra, Ranchi, 835215, Jharkhand, India; d Department of Life Sciences, School of Pharmacy, IMU University, Kuala Lumpur, Malaysia; e Institute of Pharmaceutical Sciences, J.S. University, Shikohabad, Uttar Pradesh, India; f University Institute of Pharma Sciences, Chandigarh University, Mohali, 140413, Punjab, India; g JBIT College of Pharmacy, 23 Milestone, NH-07, Chakrata Road, Shankarpur, Dehradun, Uttarakhand, 248197, India; h School of Nutrition Sciences, Faculty of Health Sciences, University of Ottawa, Ottawa, Ontario, K1N 6N5, Canada; i Department of Biomedical Engineering, Pukyong National University, Room 1317, Building A1245, Yongso-ro, Nam-gu, Busan, 48513, South Korea

**Keywords:** mRNA, Lipid nanoparticle, Exosomes, Ligand-receptor targeting, Extra hepatic delivery

## Abstract

Recent breakthroughs in mRNA therapeutics have transformed vaccine development, largely powered by lipid nanoparticle (LNP) based delivery systems. However, these systems exhibit a strong hepatic tropism, making them suboptimal for targeting extrahepatic organs such as the brain, lungs, pancreas, heart, and tumor tissues critical to non-vaccine therapeutic applications. This review explores next-generation delivery strategies designed to overcome liver centric distribution. We highlight emerging platforms, including pKa-tuned LNPs, polymeric and peptide-based carriers, exosomes, and biomimetic vesicles, along with physical enhancement techniques such as ultrasound, laser, and MRI-guided systems. Nonetheless, researchers are achieving more precise delivery to deep seated tissues by integrating these technologies with targeted ligands and responsive release mechanisms. Applications in oncology, cardiology, pulmonology, and neurology are discussed with a focus on preclinical and early clinical outcomes. Regulatory considerations, including immunogenicity, biodistribution, and manufacturing scalability, are also reviewed. Ultimately, this article presents a forward-looking perspective on engineering safe, organ specific mRNA delivery platforms beyond the liver, enabling the advancement of precision therapeutics. This review will provide a timely and comprehensive overview of innovative strategies to overcome these challenges, focusing on non-vaccine applications.

## Graphical abstract

Next-generation mRNA delivery systems engineered to overcome liver-dominant uptake. Modified carriers and targeting strategies enable efficient delivery to extrahepatic organs such as the lungs, brain, heart, pancreas, tumors, and lymph nodes.

**Figure:**
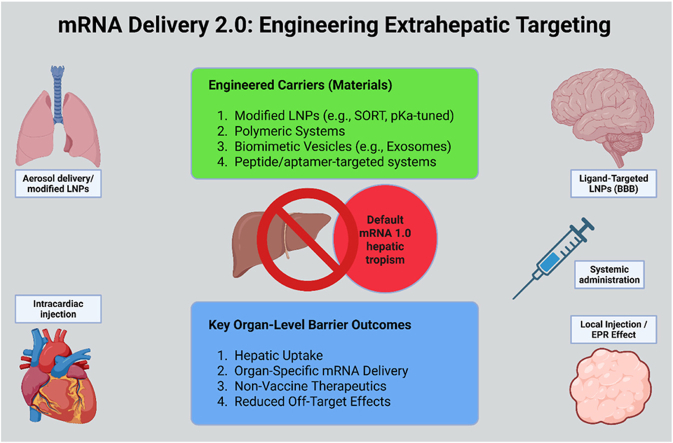

## 1 Background: mRNA therapeutics landscape

**Fig. 1:**
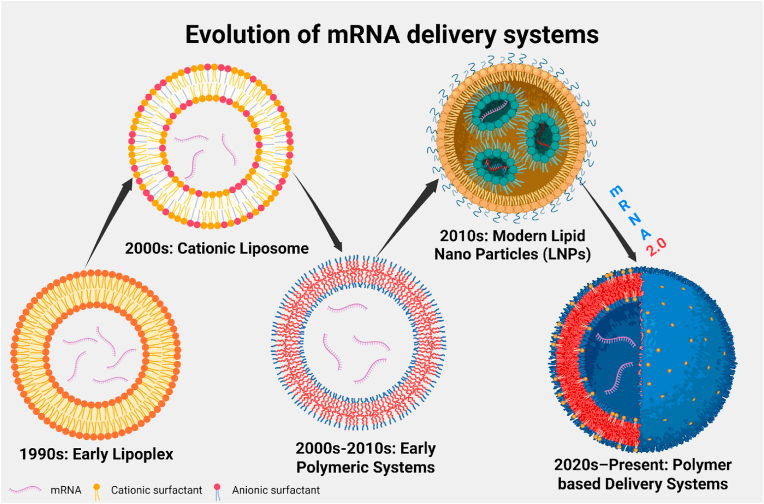
**represents a schematic illustration of a timeline of key breakthroughs in mRNA delivery (1990**–**2025).** It shows the evolution of mRNA delivery systems from early lipoplexes to modern polymer-based nanoparticles. Representative materials highlight key advances in cationic lipids, LNPs, and emerging polymers.

Messenger RNA (mRNA) therapeutics introduce a synthetic mRNA sequence into target cells, which the cellular ribosomes then translate into the encoded protein. Unlike DNA-based gene therapies, mRNA operates in the cytoplasm and does not require nuclear entry or risk genomic integration, providing a transient but tunable protein production platform. This mechanism allows mRNA to supply functional proteins that may be missing or deficient in disease, for instance, an enzyme in a metabolic disorder or an antigen in vaccine applications. Advances in mRNA chemistry have made this possible: incorporating modified nucleosides (*e.g.,* pseudouridine or 1-methylpseudouridine) into the mRNA can greatly suppress recognition by innate immune sensors and RNAse enzymes, thereby improving stability and translation efficiency and leading to excessive innate immune activation. [1,2]. If we highlight historic evolution, early efforts in the 1990s established the concept that exogenous mRNA could produce direct protein (*in vivo*). A landmark 1990 study demonstrated that injecting synthetic mRNA into mouse muscle led to expression of the encoded protein, albeit transiently and at low levels. Progress (see Fig. 1) was gradual: researchers discovered ways to stabilize mRNA molecules and avoid their degradation by ubiquitous RNases, as well as methods to dampen innate immune responses that would otherwise shut down protein synthesis. A key development (Karikó & Weissman, 2023 Nobel Prize) came in 2005 [3], when it was shown that certain modified nucleosides could be incorporated into mRNA to suppress activation of Toll-like receptors (TLR) and other sensors. By the 2010s, mRNA therapies entered clinical testing, and validation of mRNA technology was decisively demonstrated during the COVID-19 pandemic (encoding the SARS-CoV-2 spike protein) [4,5]. The first regulatory approvals in the RNA therapeutics field involved small interfering RNA (siRNA) drugs targeting liver diseases, but now mRNA has become an equally prominent modality [6,7]. Collectively, these advances have validated the clinical utility of mRNA, overcoming its historical instability with therapeutic possibilities ranging from vaccines to protein replacement and even CRISPR-based genome editing *in vivo* [[8], [9], [10]]. Despite the recent successes, first-generation mRNA delivery systems have important limitations that drive the need for further innovation. Most current mRNA therapeutics rely on lipid nanoparticles (LNPs) carriers administered intramuscularly or intravenously. These LNPs naturally tend to accumulate in the liver due to size and lipid composition, as well as uptake by the mononuclear phagocyte system, and pose a challenge for extrahepatic delivery. In fact, the clinically approved LNPs undergo rapid clearance to the liver and spleen and show limited biodistribution to other organs [1]. Another limitation is the instability of mRNA molecules. For example, the initial COVID-19 mRNA vaccines had to be kept at ultra-low temperatures to maintain potency, underscoring the inherent lability of mRNA-LNPs formulations. Early mRNA constructs caused strong interferon responses *via* sensors like TLR7/8 and retinoic acid-inducible gene (RIG-I), which produce inflammation also shut down protein translation in transfected cells. Advances such as nucleoside modification and removal of double-stranded RNA contaminants have mitigated this issue, but even current mRNA-LNPs formulations can trigger reactogenic effects in a dose-dependent manner, and repeated dosing may be limited by immune reactions against delivery components (for instance, anti-polyethylene glycol (PEG) antibodies can form, since many LNPs incorporate PEGylated lipids) [2]. Formulating LNPs at scale with consistent quality required significant process development; this was exemplified by the global effort to scale up vaccine production in 2021. In summary, the first wave of mRNA therapeutics proved the concept but came up with a set of constraints, predominant liver delivery, mRNA instability, innate immune activation, and manufacturing challenges, that limited the initial applications largely to vaccines and liver-targeted treatments. These limitations set the stage for ongoing efforts to develop “mRNA Delivery 2.0” systems with broader tissue targeting and improved performance [1,2].

mRNA 1.0 leveraged liver-tropic LNPs and local injection for vaccines and hepatocyte targets, with limited control over extrahepatic tropism, endosomal escape, and redosing. We use “mRNA Delivery 2.0” to denote platforms and workflows that simultaneously achieve: (i) extrahepatic organ targeting (via composition tuning or ligands), (ii) improved endosomal escape at tolerated doses, (iii) route engineering or device-assisted access (e.g., aerosol, intrathecal, catheter-based, focused ultrasound), and (iv) pooled, *in vivo* screening/analytics (e.g., SORT; FIND) that optimize delivery in living systems. These advances enable non-vaccine therapeutics like protein replacement, immunomodulation, and reprogramming.

## 2 Challenges in extrahepatic delivery

**Fig. 2:**
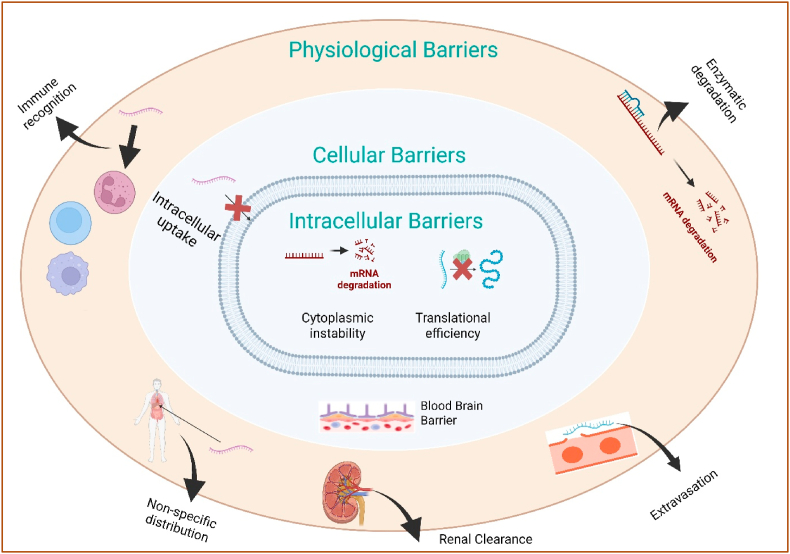
Schematic representation of various physiological, cellular, and intracellular barriers impeding extrahepatic mRNA delivery. The delivery of mRNA therapeutics to target tissues outside the liver is challenged by multiple barriers at different biological levels. **Physiological barriers** include immune recognition by immune cells, leading to clearance; enzymatic degradation of mRNA in extracellular fluids; non-specific biodistribution to off-target organs; renal clearance through the kidneys; and extravasation limitations preventing mRNA carriers from leaving the vasculature and reaching tissues. **Cellular barriers** involve inefficient cellular uptake of mRNA-loaded nanoparticles by target cells. **Intracellular barriers** further hinder therapeutic efficacy, including cytoplasmic instability caused by nucleases leading to rapid **mRNA degradation,** and poor **translational efficiency** due to suboptimal interaction with ribosomes and the translational machinery. Overcoming these multilevel barriers is critical for achieving effective and safe extrahepatic mRNA delivery for therapeutic applications.

Developing mRNA therapies for organs beyond the liver needs precise targeting and overcoming numerous biological barriers (see Fig. 2). Unlike hepatocytes, which are potentially transfected by current LNPs *via* endogenous apolipoprotein mediated uptake, successful mRNA delivery to other tissues involves multiple additional hurdles after nanoparticles (NPs) accumulation, including cellular internalization, endosomal escape, and cytoplasmic release required for functional mRNA expression. We have analyzed extrahepatic delivery across three interacting scales: systemic (protein corona, RES uptake, renal/biliary clearance), tissue/organ (default hepatic capture; blood brain barrier (BBB); mucus/ECM; disease-induced permeability), and cellular (cell entry and endosomal escape). Each platform in section 3 is discussed in terms of which scale it addresses; section 5 shows how route selection complements material design.

### 2.1 Biological barriers

Systemically delivered mRNA must survive in the bloodstream, exit the circulation at the target tissue, enter the cells of interest, and escape the endosomal compartment to reach the cytosol. Each step in this delivery cascade imposes distinct biological constraints. In the blood, naked mRNA would be degraded within minutes by RNases, so it needs to be shielded. Even when encapsulated, NPs can be opsonized by serum proteins and captured by phagocytic cells in the liver and spleen as part of the body's clearance mechanisms. One reason most intravenously administered LNPs accumulate in the liver is that the liver's sinusoidal endothelium and resident macrophages efficiently filter out a large fraction of NPs from circulation. For tissues like the lung or pancreas, which are perfused by the systemic circulation, only a small proportion of the dose might reach the capillaries feeding the target cells. Crossing the vascular endothelium is another barrier; certain organs (*e.g.,* brain) have nearly impermeable endothelial tight junctions (the BBB), whereas others, like the spleen or liver, have fenestrated capillaries that are more permissive. Following extravasation into the tissue interstitium, NPs encounter the dense extracellular matrix and, in some cases, mucus barriers that impede particle diffusion. Finally, the NPs must be taken up by the target cell (often *via* endocytosis) and then release the mRNA into the cytosol. Endosomal escape represents a critical bottleneck, as estimates indicate that only 1–2 % of the internalized NP payload successfully reaches the cytoplasm, with the rest being trafficked to lysosomes for degradation [1]. This inefficiency of endosomal escape significantly limits the dose of mRNA that ultimately becomes available for translation in the target cells [11]. Together, these biological barriers mean that delivering mRNA to organs like the lungs, heart, or solid tumors is far less efficient than delivering to the liver. Effective extrahepatic delivery systems must therefore be engineered to navigate or bypass these obstacles, for instance, by enhancing circulation time by modulating particle size and surface properties to improve tissue extravasation, and by using materials or molecules that promote endosomal release of mRNA once inside cells. Each organ presents unique anatomical and physiological challenges, as highlighted by recent reviews mapping out organ-specific barriers for non-viral gene delivery [11]. Overcoming these barriers is a central goal in the field of RNA therapeutics 2.0.

### 2.2 Targeting specificity

**Fig. 3:**
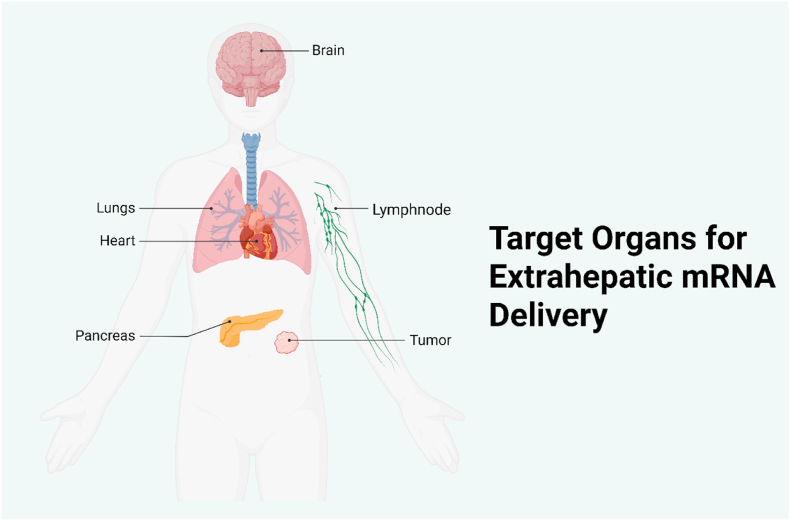
Key Extrahepatic Target Organs Routes: inhalation (lung), local injection (heart/tumors), IV/systemic (pancreas/brain).

Even if a delivery system can reach beyond (see Fig. 3) the liver, achieving specificity for the desired tissue and cell type is another critical challenge. Without some form of targeting, systemically delivered NPs may distribute broadly, leading to off-target transfection in unintended organs or cell populations. Such off-target expressions of the therapeutic protein can reduce efficacy and raise safety concerns. For example, an mRNA intended to express a hormone in pancreatic β-cells might also be taken up by liver cells or immune cells, leading to unintended or adverse effects. Targeting can be approached in two ways: (1) Passive targeting exploits the natural biodistribution tendencies of NPs to favor accumulation in certain tissues. For instance, small LNPs with certain compositions might penetrate tumorous tissue more readily or, as recently shown, incorporating specific lipids can bias LNPs towards lung or spleen uptake [11]. However, passive targeting frequently lacks precision and offers limited spatiotemporal control. (2) Active targeting involves functionalizing the NPs with ligands that bind to receptors expressed on the target cell type. In theory, active targeting can enhance uptake by desired cells, but in practice, many targeting ligands lack absolute specificity-the receptor might be present on other cells as well, leading to off-target uptake [11]. Moreover, grafting ligands onto LNPs can sometimes destabilize the particles or be masked by protein corona formation in blood. As of mid-2025, no antibody/peptide-targeted RNA nanomedicine has received full approval, though 10+ candidates (*e.g.,* DTX-0416 for solid tumors) are in Phase 2 [NCT04891584, NCT05262530] [[12], [13], [14]]. Therefore, one of the key challenges is to improve the specificity of mRNA delivery systems so that the payload is expressed predominantly in the intended cells. Strategies under investigation leverage tissue-specific targeting ligands (for example, antibodies against lung endothelium or peptides for heart tissue), as well as strategic design of the mRNA cargo itself (for instance, incorporating microRNA target sites so that the mRNA is selectively silenced in off-target cells). Another approach is regional delivery: administering the mRNA locally to the target organ (such as by inhalation for lung delivery or direct injection into a tumor or the myocardium). This can greatly limit off-target distribution by physically concentrating the therapy in the desired site. Each strategy presents distinct advantages and limitations, but all share the objective of confining therapeutic protein expression to the intended target tissue. Achieving high targeting specificity remains difficult, and often a balance must be struck; some leakage to off-target cells may be tolerable if it does not cause harm, especially if enough of the dose still reaches the target cell to have a therapeutic effect [11]. Ongoing research in NPs engineering is focused on sharpening this specificity, for example, by developing “organ-selective” LNPs and analyzing their biodistribution with advanced *in vivo* imaging and sequencing techniques [11].

### 2.3 Immunogenicity

The innate immune response to exogenous mRNA has both beneficial and detrimental effects depending on the therapeutic application. For vaccine applications, a moderate immune stimulation is desirable to help trigger an immune response. In contrast, for protein-replacement or gene therapy uses, one wants the delivered mRNA to evade immune detection to maximize translation of the therapeutic protein. Unmodified mRNA will activate pattern-recognition receptors such as TLR7 and 8 and RIG-I/MDA5, leading to production of interferons and pro-inflammatory cytokines. This not only causes systemic side effects but also induces an antiviral state that can shut down protein synthesis in transfected cells. Significant progress has been made in reducing the immunostimulatory profile of therapeutic mRNAs. As noted above, the incorporation of modified nucleosides like pseudouridine and 5-methylcytidine in place of uridine can mask the mRNA from TLRs and RIG-I, dramatically lowering interferon activation [2]. mRNA is also typically produced with a defined cap structure (Cap1) and poly(A) tail to mimic endogenous mRNA and avoid exposing uncapped 5′-triphosphate ends that strongly activate RIG-I. High-performance liquid chromatography purification of *in vitro* transcribed mRNA is used to remove any double-stranded RNA impurities (short duplex byproducts that are potent RIG-I agonists). These measures have enabled the current mRNA therapies to be administered without causing the kind of severe cytokine storms that early gene therapy attempts encountered. Nevertheless, residual innate immune sensing persists despite these advances. At higher doses, mRNA-LNPs can still trigger acute inflammatory responses; for example, the COVID-19 mRNA vaccines at high doses caused transient high fevers and flu-like symptoms in some recipients. For chronic dosing of mRNA (such as in a protein replacement therapy), repeated activation of innate immunity can blunt translation and limit redosing. Researchers are investigating additional ways to modulate immunogenicity: one idea is to use mRNA sequences that inherently produce fewer TLR-stimulatory byproducts, or to include sequences that actively suppress immune sensors. Another consideration is adaptive immune responses against the delivery system. Many LNPs include PEG-lipid for stability [15], but PEG can induce anti-PEG antibodies upon first exposure, which in subsequent doses might cause rapid clearance of the particles or complement activation (a phenomenon known as CARPA). Indeed, in some trials of LNP-based drugs, patients have experienced mild infusion reactions attributable to complement activation. Adjusting the lipid composition or pre-medicating with steroids can help manage these effects, but immunogenicity remains a concern, especially for repeated-dose mRNA therapies. Optimizing mRNA evasion of immune detection while retaining safety monitoring capacity for therapeutic efficacy (for instance, ensuring that the innate response is not completely zero in case of unwanted distribution) is an area of active investigation. Overall, while current mRNA delivery systems have greatly improved on the immunogenicity front, ensuring that the mRNA does not trigger detrimental immune activation in off-target cells or upon multiple administrations is still an important challenge for expanding mRNA treatments to new indications [2].

### 2.4 Scalability and manufacturability

The rapid development of mRNA vaccine technology in 2020–2021 showcased that mRNA therapeutics can be manufactured at scale, but it also exposed several challenges in production and distribution. Manufacturing mRNA medicine involves two primary components: the mRNA drug substance and the NPs delivery system. The mRNA is produced by *in vitro* transcription from a DNA template, a process that can be scaled in batch reactors, but the yield and quality must be carefully controlled. One challenge is ensuring purity any contaminating double-stranded RNA or residual DNA could induce immune responses or affect dose consistency. Scalable purification methods are needed to obtain highly pure mRNA, and these add to production complexity and cost. The LNPs formulation is typically formed by mixing the mRNA with lipids under precise conditions (often using microfluidic mixers or ethanol injection methods). Scaling this mixing process while maintaining uniform particle size and encapsulation efficiency presents significant technical challenges [1,16]. During the COVID, vaccine scale-up necessitated the development of large scale LNPs production processes and addressed issues like filter fouling, batch-to-batch variability in particle size, and throughput limitations of microfluidic devices. Even after production, mRNA-LNP products pose stability challenges; they are sensitive to temperature and agitation. The current vaccines have a refrigerated shelf-life of only a few months, and some require ultracold storage initially because the mRNA would degrade over time at 4 °C. Lyophilization has been explored to improve stability, with some success, but freeze-drying an LNP while preserving its structure and activity is technically challenging and requires cryoprotectants and optimization [17]. Recent research has identified specific degradation pathways and is leading to improved formulations with better shelf-life [17]. Another aspect of scalability is cost: mRNA therapies remain expensive to produce relative to small-molecule drugs, due in part to the cost of raw materials like the cap analogs, modified nucleotides, and high-purity lipids, as well as the need for cold-chain logistics. As manufacturing processes mature, production costs are anticipated to decrease, but for now, manufacturability is a barrier for some applications that would require exceptionally large doses or sustained supply. Lastly, regulatory considerations for a relatively new product class mean that manufacturing processes must meet evolving guidelines for product consistency, purity, and potency assays. Every change in a lipid component or a subtle tweak in the process might necessitate extensive characterization. The field is actively working on platform approaches, such as using the same delivery formulation for multiple mRNA products to streamline development. In summary, while the feasibility of large-scale mRNA vaccine production has been demonstrated, further innovations are needed to make manufacturing more efficient and to produce mRNA therapies that are stable at ambient temperatures for easier global distribution [17]. Improvements in lipid components and formulation methods are already being reported that could extend product stability and simplify logistics. Solving these manufacturability and scale challenges is essential for the widespread adoption of mRNA therapeutics outside of emergencies.

## 3 Advanced delivery platforms enabling extrahepatic targeting

**Fig. 4:**
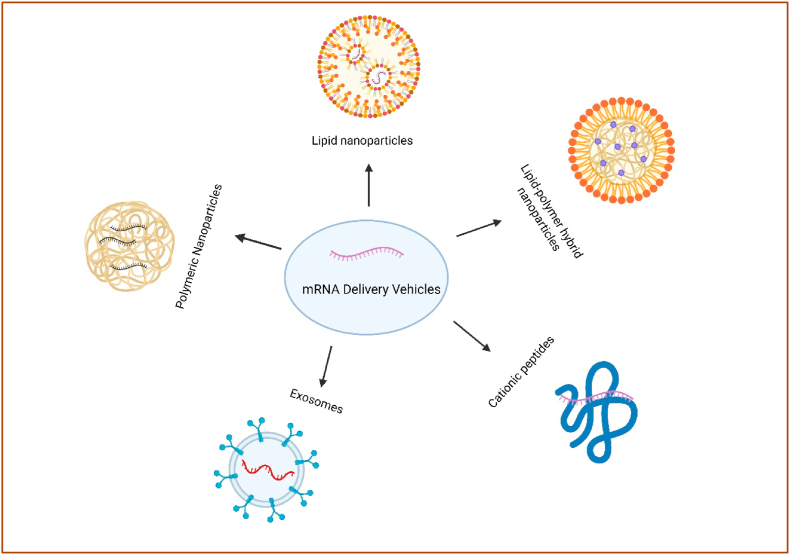
Various nanocarrier platforms employed to protect and transport mRNA to extrahepatic tissues.

Advanced delivery technologies are transforming the biodistribution profile of mRNA therapies, emphasizing the targeting of organs beyond the liver. For each class (lipid, polymer, peptide/protein, extracellular vehicles (EVs.

), hybrids). We emphasize the mechanism that solves a specific barrier (e.g., ionizable lipid pKa → endosomal escape; micelles → mucus/ECM transit; EV membranes → immune stealth; ligands → transcytosis or inflamed endothelium capture). Cross-class comparisons are summarized and shown **(see** Fig. 4 and Table 1**)**. To clarify distinctions among delivery platforms, we organize carriers by class and chronology: (3.1) lipid-based systems (ionizable/cationic LNPs; liposomes; lipoplexes), (3.2) polymeric NPs (Polylactic coglycolic acid (PLGA), Poly (β-Amino ester (PBAE), polyesters; micelles), (3.3) peptide/protein carriers, (3.4) extracellular vesicles, and (3.5) hybrids (lipopolyplexes (LPs), lipid exosome mimics). Historically, polymer-based nucleic-acid carriers predate modern LNPs; they represent a parallel technology family rather than a “next stage.” Here, “polymer NPs” include polymeric micelles, polyesters (e.g., PLGA), PBAEs, and hybrids such as LPs. Each subsection discusses design variables (pKa/ionization, degradability, size/morphology, mixing method), intracellular trafficking and endosomal escape, organ-level biodistribution, and manufacturability. *Terminologies used in this section:* Liposomes are bilayer vesicles enclosing an aqueous core; nucleic acids are typically encapsulated or complexed on the surface. Lipoplexes are electrostatic complexes of cationic lipids and nucleic acids, often lacking a continuous bilayer and forming condensed aggregates. Clinical “LNPs” are non-lamellar NPs formed by rapid mixing of ionizable lipids, helper lipid, cholesterol, and PEG-lipid; they package RNA internally and rely on ionization-driven endosomal escape rather than stable cationic charge. These distinctions matter for biodistribution and toxicity and are reflected in the references cited [[18], [19], [20]].

**Table 1:** Comparison of mRNA delivery platforms.

Delivery Platform	Typical Size	Delivery Efficiency	Liver Tropism	Extrahepatic Potential
**Naked mRNA** (no carrier)	Molecular scale (nm range)	Very low (rapid degradation; requires high doses)	None inherent (widely dispersed)	Minimal (effective only with local injection)
**Lipid Nanoparticles (LNP)**	∼50–100 nm diameter	High (efficient endosomal release; clinically validated)	High (tends to accumulate in liver by default)	Moderate (can be engineered for other organs)
**Polymeric Nanoparticles**	50–200 nm (varies)	Moderate (variable transfection efficacy)	Moderate (some liver uptake, depending on polymer)	Moderate (targeting ligands or controlled release can improve tissue specificity)
**Viral Vectors** (e.g. AAV)	∼20–100 nm (capsid size)	Very high (efficient cell entry and gene expression)	Vector-dependent (some AAV serotypes target liver)	High (can target specific organs via capsid tropism, but immunogenicity is an issue)
**Extracellular Vesicles** (exosomes)	∼50–150 nm (natural vesicles)	Experimental	Low (no strong liver tropism inherently)	Potentially high (native targeting to certain cells; can be engineered for organ-specific delivery)

### 3.1 Next-generation LNPs

Lipid-based nucleic acid delivery methods, including lipoplexes and LNPs, are the most therapeutically advanced platforms for mRNA delivery at present. Initially, lipoplexes were mostly designed for plasmid DNA delivery, whereas LNPs were refined for siRNA applications [21,22]. Nonetheless, due to the increasing interest in mRNA-based therapeutics, partly fueled by their potential applications in vaccines, cancer immunotherapy, and protein replacement therapies, these lipid-based carriers have been substantially modified and refined to efficiently encapsulate and deliver mRNA molecules. Their capacity to safeguard mRNA from enzymatic destruction, promote cellular uptake, and ensure effective endosomal escape has established them as the preferred option for mRNA administration in both preclinical and clinical applications. Multiple mRNA-loaded lipoplexes and LNPs are presently undergoing clinical studies to elicit either intratumoral production of immunostimulatory cytokines or a therapeutic or prophylactic vaccination effect. A prominent instance is the clinical trial examining the combination of mRNA-4157 with anti-programmed cell death protein 1 (PD-1) therapy, which showed a substantial 65 % decrease in distant metastases and mortality vs PD-1 treatment alone [23]. A further potential approach is chimeric antigen receptor (CAR) antigen vaccinations, referred to as CARVac [24]. A clinical trial by BioNTech utilized a combination of CAR-T cell therapy targeting the CLDN6 antigen and an mRNA vaccination encoding the same antigen (CARVac) for the treatment of solid tumors. The research demonstrated that the mRNA vaccine markedly enhanced the anticancer efficacy of CAR-T cells, highlighting the promise of mRNA-LNP platforms in augmenting immunotherapy effectiveness.

Amino lipids are essential for augmenting cellular absorption, facilitating endosomal escape, and raising the overall acceptance of LNPs. Altering the molecular structure of those lipids directly affects the efficacy and biocompatibility of the delivery method. Positively charged lipids, like 1,2-di-O-octadecenyl-3-trimethylammonium-propane (DOTMA) and 1,2-dioleoyl-3-trimethylammonium-propane (DOTAP), include alkylated quaternary ammonium groups that sustain a consistent cationic charge. The positive charge enhances electrostatic interactions with the charged phosphate backbone of mRNA, hence encouraging efficient encapsulation [25]. Nonetheless, despite their advantages, these lipids are associated with certain toxicities, such as the disruption of cellular and nuclear membranes and the production of reactive oxygen species (ROS) [26], underscoring the necessity for meticulous tuning to reconcile efficacy with safety.

To mitigate the cytotoxicity associated with permanently cationic lipids, ionizable lipids were engineered as a more benign alternative. These lipids possess pH-sensitive tertiary amine groups in their head structure, which become protonated based on the ambient pH. At normal pH, the amine groups predominantly retain a neutral charge, which diminishes interactions with biological membranes and hence lowers systemic toxicity. In acidic settings, such as during NPs manufacturing or within endosomes, the amines become protonated and acquire a positive charge. This pH-responsive characteristic improves mRNA encapsulation in acidic environments and facilitates endosomal escape following the internalization of the LNPs. The temporary cationic state of ionizable lipids in acidic compartments leads to the instability and breakdown of the endosomal membrane, promoting mRNA release into the cytoplasm [21,24,27].

Onpattro, produced by Alnylam Pharmaceuticals, was the inaugural siRNA-loaded LNPs therapeutic to obtain U.S. Food and Drug Administration (FDA) approval in 2018. It is suggested for the management of hereditary transthyretin-mediated (hATTR) amyloidosis [28]. The ionizable lipid employed in this formulation, (6Z,9Z,28Z,31Z)-Heptatriaconta-6,9,28,31-tetraen-19-yl 4-(dimethylamino) butanoate (DLin-MC3-DMA), was chosen from a collection of 56 ionizable lipids, maintaining a constant hydrophobic dilinoleyl tail while altering the head group structure. This study emphasized the significance of the apparent pKa of ionizable lipids in facilitating effective gene delivery. The effective endosomal pKa window (∼6.2–6.5) and helper-lipid driven non-bilayer phases promote endosomal destabilization; SORT (Selective Organ Targeting) adds a fifth component to bias tissue-level deposition (see Table 5) [29]. The most therapeutically advanced ionizable lipids for mRNA delivery are ALC-0315 and SM-102, utilized in the COVID-19 vaccines BNT162b2 (Pfizer-BioNTech) and mRNA-1273 (Moderna), respectively. Moderna selected the lipid SM-102 after screening 30 biodegradable ionizable lipids for their expression potential, immunogenicity, and tolerability post-intramuscular injection [29]. Moderna evaluated a range of amino lipids to create LNPs appropriate for long term delivery, to improve efficacy and minimize toxicity relative to MC3. This initiative resulted in the discovery of “lipid 5,” which exhibited enhanced protein expression and decreased toxicity in rodent and non-human primate models following intravenous administration.

Kauffman et al. further refined a formulation utilizing the ionizable lipid C12-200, originally intended for siRNA delivery. Through the optimization of the C12-200-to-mRNA weight ratio, lipid composition, and the selection of helper lipid type, they attained a formulation exhibiting up to sevenfold enhanced potency for mRNA distribution relative to its siRNA equivalent [30].

Recent evidence in ionizable lipids has potentially demonstrated the specificity and efficiency of LNP mediated mRNA delivery. Structure activity relationship data provide that subtle modifications in linker moieties profoundly impact *in vivo* delivery outcomes (Ramishetti et al. 2020) [31]. For instance, Naidu et al. (2023) demonstrated a combinatorial library of ionizable lipids that showed cell-type-specific mRNA delivery and targeted transfection in distinct immune cells. Authors also highlighted the importance of lipid architecture in modulating cellular uptake and endosomal escape mechanisms [32]. Similarly, in another study, Naidu et al. (2025) investigated ionizable amino lipids with optimized hydrazine, hydroxylamine, and ethanolamine linkers that substantially promote lung-specific mRNA delivery, providing a promising platform for treating pulmonary diseases. This lipid library allowed selective gene silencing in primary leukocytes and exhibited favourable biodistribution, low toxicity, and minimal immune activation [33]. Research indicates that the surface charge of LNPs in circulation and hence their surface composition significantly affects the formation of the protein corona, which subsequently impacts the cellular absorption mechanisms of these NPs [34]. Akinc et al. investigated the *in vivo* delivery mechanism of siRNA-loaded Dlin-KC2-DMA LNPs, demonstrating that apolipoprotein E (ApoE), a plasma protein, binds to these LNPs and functions as an endogenous targeting ligand. GalNAc-iLNPs exhibited mean particle sizes between 40 and 70 nm, aligning with the established ASGPR binding threshold for particles up to 70 nm; however, the binding of 90 nm particles was notably diminished. A mere 0.15 mol% GalNAc ligand was adequate to attain near-maximal activity. *In vivo*, PEG-shielded iLNPs exhibited no activity even at dosages reaching 3 mg/kg, whereas the inclusion of GalNAc markedly improved activity with an ED_50_ of less than 1 mg/kg. The ApoE-LNP complex is identified by low density lipoprotein receptors (LDLRs) on hepatocytes, facilitating effective liver-targeted siRNA delivery [35].

Cheng et al. developed a technique called SORT to extend delivery beyond the liver. This method incorporates a fifth element designated as the SORT molecule into the conventional four-component LNP formulation. Conventional LNPs with a nearly neutral surface charge generally demonstrate significant liver targeting following intravenous delivery [35]. Nevertheless, the integration of various SORT molecules enables organ-specific delivery. The incorporation of DOTAP, a persistently cationic lipid, redirected distribution to the lungs, whereas the addition of 1,2-dioleoyl-sn-glycero-3-phosphate (18 PA), a negatively charged lipid, enhanced preferential delivery to the spleen [36]. This highlights the potential for methodical lipid composition design to influence LNP biodistribution for targeted treatments (see Table 5). In addition to the well-established SORT for modulating LNP, recent preclinical practices have demonstrated that precise tuning of lipid component proportions within LNP formulations, for instance, Rampado et al. (2024) reported that fine adjustments in the ratios of ionizable lipids, phospholipids, cholesterol, and PEG lipids. They enhanced colon targeting and transfection efficiency of mRNA therapeutics, which highlighted the critical influence of lipid composition on NPs' physicochemical properties, cellular uptake, and tissue accumulation profiles and promoted achieving SORT beyond the liver [37].

LNPs are fundamental to mRNA delivery owing to their shown efficacy and therapeutic effectiveness. Nonetheless, novel tactics are progressively concentrating on adjusting LNP composition to modify biodistribution and improve therapeutic efficacy. This strategic shift is prompted by biodistribution, the direct influences of the site, and the efficacy of LNPs in cargo delivery. Conventional LNPs predominantly concentrate on the liver, hence restricting their therapeutic use to hepatic disorders. By modification, researchers can route LNPs to extrahepatic regions, improve cellular absorption, and reduce off-target effects. An important development in this field is the progression of ionizable lipids, which facilitate endosomal escape while reducing systemic toxicity. Furthermore, conventional PEGylation, previously prevalent for prolonging circulation half-life undergoing reassessment. Investigations are underway into alternatives or supplements to PEG, including various hydrophilic surface coronas, to enhance tissue penetration and diminish immune system recognition [38]. One of the most significant advancements in LNP design is SORT, a method that employs customized lipid components to direct NPs to particular organs, including the lungs or spleen. This approach extends the therapeutic scope of LNPs beyond the liver, which has been their conventional target. Numerous clinical-stage formulations already utilize advanced delivery technologies, showing potential in fields such as vaccinations, cancer treatment, and gene editing.

PEG lipids were developed to enhance colloidal stability and inhibit particle aggregation during circulation and storage. These comprise PEG, a hydrophilic polymer deemed safe for human application, conjugated to lipid tails that incorporate into the LNP surface [39]. The molecular weight of PEG and the configuration of the lipid tail both affect the physicochemical and biological properties of LNPs. PEGylation diminishes protein adsorption (opsonization), restricts clearance by the reticuloendothelial system (RES), and eventually prolongs circulation duration [40]. Due to the absence of an aqueous core in LNPs, PEG-lipids predominantly appear on the surface, influencing particle size and delivery efficacy [41].

Kulkarni et al. employed cryo-transmission electron microscopy (TEM) to illustrate that augmenting PEG concentration decreases particle size while concurrently reducing cellular absorption, resulting in diminished overall delivery efficacy [26,42]. Consequently, PEG lipid molar ratios of 1–3 % are typically used to optimize stability and efficiency. PEG lipids form a hydrophilic shield that prevents particle aggregation, serum protein opsonization, and bloodstream circulation. Low quantities (<1 %) of PEG lipids may not provide enough steric stability, causing particle aggregation and fast clearance. However, high PEG (>3 %) can reduce gene transport effectiveness by inhibiting cellular absorption and endosomal escape [43].

Despite these advantages, PEGylation is linked to two notable disadvantages. The initial phenomena are accelerated blood clearance (ABC), characterized by the fast elimination of repeated doses of PEGylated NPs from circulation [44]. Second is complement activation-related pseudoallergy (CARPA), an immunological response elicited by anti-PEG antibodies [45,46]. These antibodies are generally produced following initial exposure to PEG, commonly via a type 2 T cell-independent immunological response. Upon initial interaction, PEGylated NPs engage B cells in the marginal zone of the spleen, resulting in the synthesis of anti-PEG antibodies. Upon additional exposures, circulating antibodies attach to PEG and initiate the complement cascade, resulting in opsonization by C3 fragments and enhanced phagocytosis by Kupffer cells, culminating in expedited clearance [46].

Furthermore, complement activation results in the release of anaphylatoxins, including C3a, C4a, and C5a, which activate immune cells to secrete inflammatory mediators. Anaphylatoxins stimulate mast cells, hence leading to anaphylactic symptoms [26,47].

In response to these concerns, researchers are diligently investigating alternatives to PEG or devising techniques to mitigate PEG related immunogenicity while preserving the advantageous pharmacokinetic characteristics of LNPs formulations. For instance, Yao et al. (2022) demonstrated various PEG alternatives, including poly(N-vinylpyrrolidone), polyacrylamides, polybetaines, poly(2-oxazoline) s, polyesters, and polysarcosine, highlighting their synthesis to biomedical applications in nanocarriers, hydrogels, and surface coatings [48]. Moreover, Berger et al. (2023) investigated that amphiphilic poly(N-methyl-N-vinylacetamide) (PNMVA) derivatives, specifically DSPE-PNMVA, potentially integrate into lipid membranes, prevent protein corona formation, and exhibit stealth properties comparable to DSPE-PEG. Unlike PEG, DSPE-PNMVA revealed no adverse effects on cellular uptake or endosomal escape, and suppressed immunological reactions, hence emphasizing the potential of such PEG alternatives [49].

### 3.2 Polymeric NPs

Despite considerable progress in mRNA delivery via LNPs, alternate delivery techniques are being thoroughly explored to mitigate certain intrinsic constraints. Significant problems related to LNPs encompass dose-limiting toxicity and a pronounced propensity for hepatic accumulation, so constraining their use for targeting alternative organs and tissues [50]. To address these challenges, researchers have utilized synthetic polymers specifically cationic and ionizable types as viable alternatives for nucleic acid transport.

Among these, PBAEs and PLGAs have attracted significant interest. These polymers provide numerous benefits, such as Ionizable PBAE/polyester micelles form compact polyplex-in-micelle structures that aid mucus/ECM transit and enable triggered unpacking in endosomes, degradable backbones that mitigate chronic toxicity; relative to previous-generation materials like polyethylenimine (PEI), which, although exhibiting great transfection efficiency, is recognized for its considerable cytotoxicity [51,52]. PBAEs have exhibited effective mRNA delivery capabilities while preserving a positive safety profile, rendering them appealing candidates for further advancement. Likewise, PLGAs, currently sanctioned for clinical application by providing the additional advantage of biodegradability and adjustable release kinetics, however, PLGA itself is not cationic polymers and thus lack the inherent ability to complex with negatively charged RNA molecules. Instead, PLGA's utility in RNA delivery is mainly from its hydrophobic properties, which aid in NPs assembly and encapsulation of therapeutic cargo, and from its well-established safety profile. To address the lack of positive charge needed for RNA complexation, PLGA is often combined with cationic or ionizable polymers such as PBAEs, which offer efficient nucleic acid binding and improved transfection, while maintaining biodegradability and low toxicity [53,54]. As research advances, these polymer-based systems may enhance or potentially exceed LNPs in specific therapeutic applications, broadening the range and accuracy of mRNA-based therapeutics.

Zhang et al. formed a NPs system based on polymers, specifically PBAE, poly-glutamic acid (PGA), and di-mannose moieties. This system contained mRNAs that encode transcription factors capable of transforming tumor-associated macrophages (TAMs), which often display tumor-promoting characteristics, into cells with an anti-tumor phenotype. This approach's therapeutic potential was evidenced in preclinical models of melanoma, glioblastoma, and ovarian cancer [52]. In a research study, pulmonary melanoma metastasis therapy decreased lung tumor burden by 8.7-fold (40 versus 419 nodules) and enhanced survival by 1.3-fold. In the PDG glioma model, tumors exhibited significant macrophage infiltration (32.8 % compared to 3.7 % in controls). NPs individually partially postponed growth, prolonging survival by 5 days; however, their combination with radiation more than doubled lifespan to 52 days compared to 25 days in controls, underscoring significant synergistic efficacy [56].

Huang and colleagues similarly developed a NPs formulation utilizing a polymer with ortho-hydroxy tertiary amine (HTA) groups in conjunction with cholesterol. These polymeric NPs demonstrated considerable anticancer efficacy *in vivo* against the B16-OVA melanoma model. Mice immunized with PHTA-C8/mOVA exhibited a marginal reduction in tumor growth relative to the PBS-treated group, but PHTA-C18/mOVA therapy resulted in prolonged tumor suppression. The PHTA-C18/mOVA showed 87 % tumour suppression efficacy, compared to PHTA-C8/mOVA, *i.e.,* 36 %. The largely stable body weights across all groups suggested that PHTA-based polymeric mRNA vaccinations were well tolerated and exhibited no evident toxicity [50].

Liu et al. introduced a novel approach, converting cationic polymers previously ineffectual for *in vivo* mRNA delivery into zwitterionic phospholipid polymers (ZPPs). The optimized ZPPs attained protein expression levels up to 39,500 times more than their cationic precursors, thus addressing the *in vivo* delivery limitations of the parent polymers. PA6-4P14 significantly facilitated mRNA translation in both the spleen and lymph nodes, indicating the extensive potential of the zwitterionic phospholipidation approach for enhancing polymer-based nucleic acid therapies and immunotherapy applications [51].

These examples demonstrate how rational polymer design can expand the biodistribution and therapeutic potential of mRNA delivery devices beyond the constraints of conventional LNPs.

### 3.3 Peptide and protein-based carriers

Lipid-based and polymeric NPs have not been the sole platforms investigated for mRNA administration; peptides have also surfaced as viable options owing to their intrinsic biocompatibility and functional flexibility. Cell-penetrating peptides (CPPs) and protamines are among the most extensively researched peptide-based delivery strategies [55]. These peptides can enhance the cellular uptake of mRNA by multiple methods, including membrane translocation and endocytosis, while also safeguarding the mRNA from enzymatic destruction. Their comparatively uncomplicated structure, ease of synthesis, and potential for functionalization render peptide-based systems appealing options for non-viral gene delivery.

Protamines were among the initial substances examined for mRNA complexation. These diminutive (∼4 kDa), arginine-rich peptides can condense mRNA into NPs with an average diameter of around 300 nm. Protamines are recognized for their ability to activate the immune system via TLR7 and TLR8. Arginine-rich CPPs enhance membrane interaction and endosomal destabilization; in hybrids, they raise cell entry while carriers provide serum stability. Although immunogenicity is generally unfavorable in drug delivery systems, it can be beneficial in particular contexts, such as cancer immunotherapy and vaccinology [56,57].

CureVac utilized this ability to create the RNActive™ platform, which integrates modified mRNA (modRNA) with protamines and has undergone clinical trials for prostate and non-small cell lung cancer.

CPPs are primarily composed of short amino acid sequences, usually fewer than 40 residues, that enable translocation across cellular membranes. CPPs frequently display either cationic or amphipathic properties. Cationic CPPs, abundant in arginine, histidine, and lysine, engage in electrostatic interactions with negatively charged cellular membranes. Amphipathic CPPs comprise both hydrophilic and lipophilic amino acids, facilitating membrane penetration via structural alignment and partitioning [58]. CPPs have been extensively utilized in siRNA distribution, either independently or in conjunction with other nanocarriers, to impede tumor progression in diverse preclinical models. When used independently, CPPs might form electrostatic complexes with negatively charged siRNA, enhancing cellular uptake in tumor cells; however, challenges like endosomal entrapment and serum instability may restrict their efficacy when administered alone. To mitigate these constraints, CPPs are frequently included in hybrid delivery systems such as liposomes, polymeric NPs, or inorganic nanocarriers, where they augment cellular uptake while the carrier offers protection and regulated release. Preclinical investigations in diverse cancer models have shown that CPP-mediated siRNA delivery can efficiently silence oncogenes, inhibit tumor proliferation, and potentially overcome treatment resistance [59,60].

Nonetheless, their utilization in mRNA delivery remains somewhat constrained. Chen et al. developed a micelle-inspired delivery system utilizing PEG, PNIPAM (poly(N-isopropylacrylamide)), and cyclic Arg-Gly-Asp (cRGD) peptides. The incorporation of cRGD improved tumor targeting and mRNA expression *in vivo*. The *in vitro* findings confirmed the efficacy of redox crosslinking and an intermediate hydrophobic barrier in enhancing systemic retention and protecting against enzymatic degradation. cRGD-functionalized nanoformulations demonstrated extended circulation in the bloodstream relative to non-cRGD formulations, indicating their potential for systemic applications. *In vivo* test results showed significant gene expression variation in tumors, particularly in the cRGD-PEG/PNIPAM-Plys (SH) group. The results confirmed the strategies of designing suitable architecture to safeguard the mRNA payload, ensure persistence in the bloodstream, and utilize surface-decorated ligand motifs for improved accumulation at the tumor site and subsequent mRNA expression in the targeted tumor cells [61].

Van den Brand et al. conducted a study employing CPP PepFect14 to develop the CPP-mRNA NPs and assessed their intraperitoneal transfection efficacy against Lipofectamine MessengerMAX (LipMM) in a murine model of ovarian cancer. The *in vivo* study revealed that PF14 NPs expressed protein across various cell types in tumor-associated tissue better than lipid-based transfection agents. Localization to the peritoneal cavity and mRNA translation in various cell groups were confirmed using primary ovarian cancer explants, indicating that transiently reducing the tumor microenvironment through protein expression, as ovarian cancer is usually restricted to the peritoneal cavity [62].

Tateshita et al. utilized a lipoplex-like nanocarrier containing the KALA peptide to formulate an *ex vivo* dendritic cell-based cancer vaccination. This method enabled effective mRNA transport into dendritic cells, underscoring the promise of CPPs in immunotherapeutic approaches. Proteomic analysis indicates that ssPalmM-KALA transfection activates a translation down-regulatory mechanism, while ssPalmE-KALA does not. OVA-specific cytotoxic T lymphocyte activity was induced *in vivo* by immunization with BMDCs pre-transfected with ovalbumin (OVA) encoding mRNA. In parallel, the immunization showed substantial anti-tumor effects against a model tumor expressing the OVA protein [63].

These examples highlight the increasing interest in peptide-based systems for mRNA delivery, particularly in the realms of cancer treatment and immunization [64].

### 3.4 Extracellular vesicles/exosomes

Exosomes, a category of EVs, measure between about 40 and 120 nm in size. They are produced by the inward budding of the endosomal membrane, resulting in the formation of multivesicular bodies (MVBs). The MVBs ultimately merge with the plasma membrane, discharging exosomes into the extracellular milieu. Exosomes inherently transport nucleic acids and proteins, promoting intercellular communication among diverse tissues [65].

Exosomes, owing to their biological origin, feature a lipid bilayer that provides enhanced biocompatibility and reduced immunogenicity relative to synthetic drug delivery vehicles. Besides their safety profile, exosomes possess intrinsic targeting capabilities, can traverse physiological barriers, and adhere to natural intracellular trafficking pathways. The distinctive benefits of exosomes and bioinspired delivery platforms have generated increasing interest as viable options for therapeutic applications [66,67]. Despite the intriguing promise of exosomes as natural mRNA delivery systems, the research remains predominantly investigational and encounters numerous technical challenges. A significant problem is the efficient loading of big molecules, such as mRNA, into exosomes. Although conventional bulk electroporation methods have effectively included short nucleic acids like siRNAs and miRNAs, this technology is ineffective for mRNA delivery.

In response, different methodologies have been devised. Yang et al. presented an innovative electroporation method termed cellular-nanoporation. This method involves transfecting cells with plasmid DNA and subjecting them to a short electrical pulse that induces the release of exosomes containing the transcribed mRNA. This method resulted in a 50-fold enhancement in exosome formation and a 100-fold augmentation in mRNA loading relative to bulk electroporation [68].

Tsai et al. utilized a technique that involved complexing mRNA with cationic lipids and subsequently incubating it with isolated exosomes to enhance cargo transfer [69]. Furthermore, advanced genetic engineering techniques have resulted in the development of systems such as EXOsomal transfer into cells, wherein mammalian cells are modified to generate exosomes that encapsulate specific mRNAs via an RNA packaging process. This approach improves exosome yield and optimizes targeted delivery efficiency. The modified exosome production enhancers augmented vesicle secretion by 3–5 folds, whereas RNA-packaging mechanisms enhanced cargo loading efficiency by 20–30 folds. *In vitro*, exosomes loaded with catalase mRNA decreased oxidative stress in neural cells, reducing ROS levels. *In vivo*, implanted producer cells released exosomes containing catalase mRNA that traversed to the brain, where they protected dopaminergic neurons in a Parkinson's mice model, decreasing neuronal loss by 30–40 % and enhancing motor performance with a 1.5-fold increase in rotarod latency [70].

Maugeri et al. employed the inherent endocytic routes of LNPs to substitute them with exosomes as delivery vehicles. When injected *in vivo*, these exosomes efficiently carried mRNA and elicited reduced levels of inflammatory cytokines compared to conventional LNPs, suggesting a potentially safer delivery profile. The research indicated that plasma hEPO protein concentrations generated by LNPs were 6–8 times greater than those produced by endo-EVs. In addition, endo-EVs consisted of a three-fold reduced amount of ionizable lipid compounds critical for LNP production but potentially toxic per mRNA compared to LNPs. The plasma expression levels of eight distinct cytokines in mice validated that endo-EVs induce a reduced inflammatory cytokine response compared to LNPs when administering an equivalent dose of hEPO mRNA to animals [71].

Despite these developments, exosome-based delivery continues to encounter substantial obstacles, particularly in terms of large-scale production, standardization, and clinical translation. Recent advancements, exemplified by Tsai et al.'s development of an exosome-based mRNA vaccine for SARS-CoV-2, which effectively induced both cellular and humoral immune responses following intramuscular delivery, underscore the increasing promise of this platform. The research results revealed that the intramuscular delivery of exosome-encapsulated SW1 and LSNME mRNAs in C57BL/6J mice produced a dose-dependent and sustained antibody response against SARS-CoV-2 Spike and Nucleocapsid proteins, with reactivity maintained during the 3-month duration of the trial. The antibody levels targeting the N protein were moderate, aligning with LSNME's strategy to elicit cellular rather than humoral protection predominantly [72]. To address the inherent constraints of synthetic NPs and exosomes, researchers are progressively investigating hybrid delivery systems that amalgamate the benefits of both. EV surface proteins engage native trafficking and reduce innate sensing; loading large mRNAs remains the bottleneck, motivating EV-hybrid strategies (discussed in section 3.5) [73,74]. Extracellular vesicle-based hybrid systems have been extensively reviewed recently and signify a promising horizon for next-generation mRNA therapies.

### 3.5 Hybrid systems

**Fig. 5:**
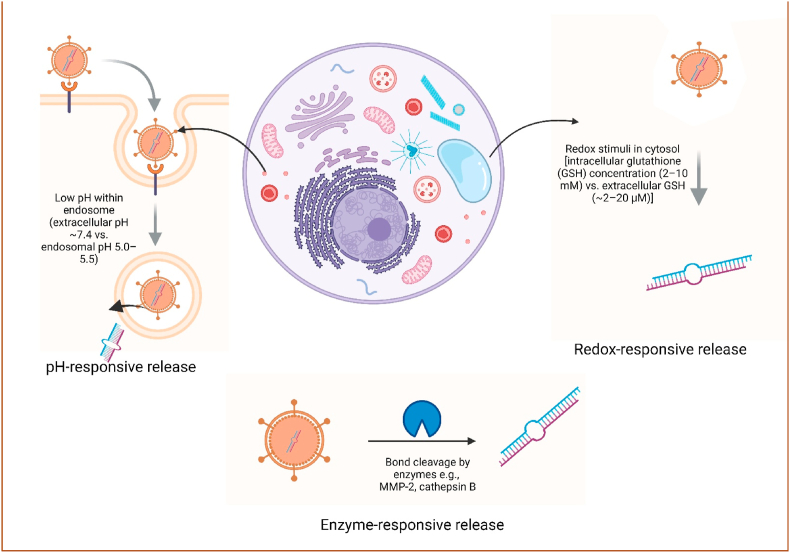
depicts only the "pH/redox/enzyme response to release" but does not label specific stimulus conditions (e.g., pH 5.5 vs 7.4, GSH concentration). Various mechanisms have been represented by which engineered nanocarriers achieve controlled release of mRNA cargo inside target cells by responding to specific intracellular stimuli. **pH-responsive release:** After receptor-mediated endocytosis, the delivery vehicle enters the endosomal compartment, where the acidic microenvironment (low pH) triggers structural changes in the carrier, facilitating endosomal escape and mRNA release. **Redox-responsive release:** Once in the cytosol, high concentrations of **reducing agents such as glutathione** induce cleavage of redox-sensitive linkages within the nanocarrier, releasing the encapsulated mRNA. **Enzyme-responsive release:** Certain delivery systems are designed with linkers or coatings cleavable by intracellular enzymes (e.g., proteases, esterases), ensuring cargo liberation only upon enzymatic activation.

Hybrid nanocarriers comprising lipid polymer particles (LPP), lipopolymer blends, or lipid-exosome hybrids leverage the strengths of individual components to achieve superior performance. Stimuli-responsive systems (see Fig. 5), sensitive to pH, redox conditions, or enzymatic activity, allow on-site release of mRNA, improving therapeutic index. Innovations like theranostic platforms combine imaging with therapy, while dual payload systems (e.g., mRNA + siRNA or immunomodulators) offer synergistic benefits for complex diseases, including cancer and autoimmunity [75]. Researchers have developed hybrid systems, known as LPs, to leverage the synergistic benefits of polymeric and lipid-based delivery platforms. These systems integrate the superior nucleic acid condensation efficiency and adaptability of polymers with improved stability, biocompatibility, and protective lipid shell characteristic of LNPs. Such combinations have been demonstrated to enhance the serum stability and transport effectiveness of polymer-based NPs [76].

Persano et al. developed a lipopolyplex mRNA vaccine by initially complexing mRNA with PBAE and subsequently encapsulating the resultant binding within a lipid shell. This formulation, when fed to mice with B16-OVA tumors, achieved a remarkable 90 % reduction in tumor nodules, indicating significant antitumor activity. Core shell mRNA vaccine penetrates dendritic cells by macropinocytosis and induces interferon-β and interleukin-12 *via* TLR7/8 signaling, demonstrating inherent adjuvant characteristics. The vaccine improved dendritic cells' antigen presentation, and lipopolyplex mRNA reduced tumor nodules by over 90 % in a lung metastatic B16-OVA tumor model. These data show that the core–shell design is a promising mRNA vaccination platform [77]. Guevara et al. enhanced this methodology by integrating α-Galactosylceramide (α-GalCer), a powerful immunoadjuvant, with PBAE-based LPPs containing therapeutic mRNA. In the B16F10 melanoma model, these LPPs elicited superior dendritic cell maturation, augmented cytotoxic T lymphocyte (CTL) responses, and increased survival rates relative to conventional LNPs, emphasizing the immunotherapeutic potential of LPPs. The research results revealed that a multi-LP vector for α-GalCer/TRP2-mRNA codelivery dramatically increased the induction of antigen-specific CD8^+^ T cells both systemically and intratumorally. Similarly, multi-LP/α-GalCer + TRP2-mRNA vaccination effectively induces humoral immune responses *in vivo*, leading to high levels of antigen-specific IgG1, IgG2b, and IgG2c. In mice vaccinated with multi-LP/α-GalCer + TRP2-mRNA, the IgG2c/IgG1 ratio was significantly higher than in mice immunized with NPs loaded with antigen-mRNA. This suggests that combining mRNA-based vaccine with αGalCer can increase Th1 response and alter the Th1/Th2 balance [78]. Moignic et al., designed an advanced LPP by initially complexing mRNA with PEGylated, histidinylated polylysine, subsequently encapsulating it within a lipid layer functionalized with α-D-mannopyranoside, a component recognized for its ability to target antigen-presenting cells. The mRNA-loaded lipid polymer particles exhibited therapeutic efficiency in three distinct mouse tumor models, underscoring their extensive utility in cancer treatment. A significant suppression of tumor growth was observed in 6 out of 9 mice, compared to 1 out of 10 mock (PBS) mice and 3 out of 9 E7-coding MN-LPR-vaccinated animals, resulting in a considerable long-term survival rate of 55 %. Notably, non-coding sspolyU triMN-LPR, unlike non-coding sspolyU MN-LPR, was capable of eliciting certain anti-tumor responses, resulting in a survival percentage of approximately 30 %. The non-antigen specific anti-tumor action may be elucidated by the inflammatory (or adjuvant) properties of triMN-LPR [79].

#### 3.5.1 Biomimetic vector nanocarriers

Erythrocyte membrane-camouflaged NPs (RBC-NPs) exemplify bioinspired nanocarriers engineered for immune evasion and extended systemic circulation. Coating synthetic NPs cores with natural erythrocyte membranes endows these carriers with “self” identifiers like CD47, thus evading phagocytic clearance. The native membrane safeguards the encapsulated medication from oxidative stress and enzymatic breakdown, hence improving stability against ROS. These attributes facilitate prolonged blood retention, less immune recognition, and enhanced delivery efficiency for therapeutic and diagnostic agents alike [80]. Hu et al. developed the Erythrocyte membrane-camouflaged polymeric NPs injections of mice with fluorophore-loaded NPs demonstrated an enhanced circulation half-life for erythrocyte-mimicking NPs compared to control particles coated with advanced synthetic stealth materials. The biodistribution investigation indicated substantial particle retention in the bloodstream 72 h post-injection. The transfer of natural biological membranes, their associated proteins, and accompanying functions to the surfaces of synthetic particles constitutes a distinctive method in NPs functionalization [81]. Bacterial outer membrane vesicles (OMVs) and membrane-coated NPs utilize inherent bacterial components to facilitate effective mucosal adherence, mucus infiltration, and immunological stimulation. OMVs, produced spontaneously by Gram-negative bacteria, encompass lipopolysaccharides and outer membrane proteins that can engage with human mucosal surfaces or biofilms, rendering them especially efficacious for pulmonary, gastrointestinal, and intranasal administration. Their intrinsic immunogenicity can be altered via detoxification or genetic modification, facilitating regulated immune activation in vaccination or cancer immunotherapy applications. [82]. Nanocarriers generated from cell membranes of cancer cells, macrophages, or leukocytes utilize homotypic recognition and immune-mimetic properties for targeted distribution. Cancer cell membrane-coated NPs can selectively concentrate at tumor sites by homotypic adhesion; however, macrophage- or neutrophil-mimetic systems can penetrate inflamed endothelium to deliver treatments to immune-privileged or inflammatory tissues [83].

## 4 Targeted tissue applications

With continued advances addressing the challenges above, mRNA delivery is expanding to a variety of specific tissues beyond the liver. Newly developed delivery strategies and formulations are enabling mRNA therapeutics to tackle diseases in organs such as the lungs, brain, pancreas, heart, and even tumors. Below, we discuss targeted mRNA applications in these tissues, including the therapeutic rationale and progress to date in overcoming delivery barriers **(See** Fig. 3 **for organ mapping)**.

### 4.1 Lung

The lungs present an attractive yet challenging target for mRNA therapy. On one hand, the lungs are accessible *via* inhalation routes, allowing for local delivery of mRNA formulations (*e.g.,* aerosolized NPs) that can act directly at the diseased site while minimizing systemic exposure [11]. On the other hand, the respiratory tract has formidable defenses. The airways are lined with mucus and ciliated cells that trap and clear particulates, and alveoli contain immune cells ready to engulf foreign matter [11]. Furthermore, aerosolized particles must have an optimal size and deposition profile to reach the deeper lung regions without being exhaled or stuck in the upper airways [11]. Despite these challenges, recent research has led to significant advances, particularly in developing inhaled mRNA therapies for diseases like cystic fibrosis. A pivotal application is in cystic fibrosis (CF), a genetic disease caused by mutations in the CFTR chloride channel. CF primarily affects the lungs with thick mucus and recurrent infections. An inhaled mRNA therapy for CF aims to deliver correct copies of CFTR mRNA to lung epithelial cells, restoring production of functional CFTR protein regardless of the patient's mutation. A recent Phase 1/2 clinical study evaluated an aerosolized LNP encapsulating codon-optimized CFTR mRNA (called MRT5005) in adults with CF (see Table 2). The mRNA was administered *via* nebulizer at multiple dose levels. Interim results indicated that inhaled CFTR mRNA (MRT5005) was generally well tolerated, with dose-related transient fever and manageable hypersensitivity reactions [10]. Over 28 days, no serious safety signals emerged, supporting the feasibility of repeated inhaled dosing [10]. However, the study did not demonstrate consistent improvements in lung function (FEV_1_), and direct evidence of translated CFTR protein in human lung tissue was not reported; thus, the data support feasibility/safety rather than clinical efficacy or confirmed in-situ protein production. In parallel, multiple inhaled mRNA programs are refining dose and formulation, including VX-522 (Vertex/Moderna; inhaled CFTR mRNA; NCT05668741) [84] and ARCT-032 (Arcturus; inhaled mRNA therapy; NCT05712538) [85], which report ongoing single- and multiple-ascending-dose cohorts. Beyond CF, mRNA delivery to the lung is being explored for other pulmonary diseases. For instance, pulmonary fibrosis, a progressive scarring of the lungs, could potentially be treated by mRNA encoding regenerative or antifibrotic factors. Preclinical studies in lung fibrosis models have shown that delivering mRNAs for certain beneficial proteins can ameliorate disease. One notable target is hepatocyte growth factor (HGF), a protein with anti-apoptotic and tissue-repair functions. In a mouse model of pulmonary fibrosis (induced by bleomycin injury), researchers used NPs to deliver an HGF gene payload to the lungs [86]. The treatment led to increased HGF levels in lung tissue and significantly reduced indicators of fibrosis and cell death, although a substantial fraction of the dose still went to the liver due to first-pass uptake after systemic delivery [87,88]. Preliminary evidence suggests that if lung-targeted delivery can be improved, mRNA-based expression of factors like HGF or keratinocyte growth factor (KGF) may promote alveolar epithelial regeneration [89,90] and counteract fibrotic processes. Another emerging area is the use of mRNA for acute lung injuries. In severe COVID-19 or acute respiratory distress syndrome (ARDS), an mRNA therapy delivered to the lung could, in theory, provide a therapeutic protein (for example, an anti-inflammatory cytokine or a soluble receptor to neutralize inflammatory signals) right at the site of injury. While no such therapy has reached clinical trials yet, preclinical research is examining possibilities like mRNA-encoded IL-10 (an anti-inflammatory cytokine) to dampen cytokine storms in ARDS, or soluble ACE2 to act as a decoy for SARS-CoV-2. Preclinical studies illustrate the concept: lung-targeted LNP-mRNA can deliver immunomodulatory proteins that attenuate inflammatory injury (e.g., sPD-L1 mRNA reducing ARDS-like pathology in mice) [91]. Although IL-10 is a well-validated anti-inflammatory effector in ALI/ARDS (with aerosol protein IL-10 improving outcomes in murine ALI), direct IL-10 mRNA delivery to the lung remains at the preclinical/early-stage concept level [92]. Separately, mRNA-encoded soluble ACE2 decoys delivered IV or by inhalation neutralize SARS-CoV-2 in models, supporting the feasibility of decoy receptor strategies for viral lung disease [93,94]. The lung's accessibility makes these ideas plausible if delivery vehicles can surmount the physical barriers. To that end, inhalable NPs are being optimized-for example, engineering bio-inspired carriers that can penetrate mucus or using dry powder formulations that aerosolize efficiently [11]. In summary, the lung is a prime target organ where mRNA therapeutics could directly correct genetic disorders like CF or locally treat acquired pulmonary diseases. Early clinical studies have established safety, and the next steps will be demonstrating clinical efficacy as delivery technologies improve. The ability to administer mRNA non-invasively *via* inhalation could enable the development of convenient, repeatable lung therapies in the future.

**Table 2:** Key extrahepatic mRNA therapies in development.

Therapy (mRNA)	Target (Disease)	Delivery Route	Status (Phase)
**CFTR mRNA** (MRT5005)	Lung (Cystic Fibrosis)	Inhaled (nebulized)	Phase 1/2 trial
**VEGF-A mRNA** (AZD8601)	Heart (Ischemic Heart Disease)	Direct myocardial injection	Phase 2a completed
**Neoantigen mRNA Vaccine** (mRNA-4157)	Immune (Post-cancer, melanoma)	Intramuscular injection (vaccine)	Phase 2b trial
**IL-2 Mutein mRNA** (mRNA-6231)	Immune (Autoimmune disorders)	Subcutaneous injection	Phase 1 trial
**Tri-cytokine mRNA** (mRNA-2752)	Tumor (Solid tumors)	Intratumoral injection	Phase 1 trial

### 4.2 Brain

The brain has historically presented a formidable barrier to systemic therapies due to the presence of the BBB, a tightly regulated endothelial barrier that prevents most macromolecules and NPs from entering the brain parenchyma. Yet neurological disorders are among the most devastating and prevalent diseases, and there is great interest in applying mRNA technology to address unmet needs in this area. Potential neurological applications of mRNA include neuroprotective therapies for neurodegenerative diseases, stroke treatments, and even cancer therapies for brain tumors. Achieving delivery to the brain requires special approaches. One strategy is direct administration into the central nervous system-for example, *via* intracerebral, intraventricular, or intrathecal injection. Direct injections can sidestep the BBB, but it is invasive and often only practical in certain settings. Another strategy is to engineer delivery systems that can cross the BBB after intravenous administration. Recent advances in nanotechnology are offering promising solutions. For instance, researchers have designed novel ionizable LNPs that incorporate “BBB-crossing” moieties (see Table 3). In one 2025 study, a library of structurally modified lipids was screened and yielded a LNP formulation capable of ferrying mRNA across the BBB in mice at appreciable levels. This optimized LNP, when given through IV, carried mRNA to the brains of mice and achieved functional mRNA expression in neurons and astrocytes across the brain, representing a notable advancement over conventional LNP formulations [9]. The formulation was well tolerated in animals and even showed the ability to deliver mRNA to *ex vivo* human brain tissue. These advances substantiate the feasibility through NPs engineering; it may be possible to penetrate the BBB or exploit mechanisms like receptor-mediated transcytosis to enter the brain. In terms of therapeutic targets, neurotrophic factors are a compelling category for mRNA delivery. Many neurodegenerative conditions are associated with loss of supportive growth factors. For example, in Parkinson's disease models, restoring glial cell line-derived neurotrophic factor (GDNF) or similar factors has shown neuroprotective effects; however, protein delivery or gene therapy efforts for GDNF have struggled with distribution in the brain. mRNA could offer a transient, regulatable way to boost neurotrophic factor levels. A recent preclinical study tackled Alzheimer's disease by delivering mRNA encoding brain-derived neurotrophic factors (BDNF), a protein that supports neuronal survival and function. Researchers formulated BDNF mRNA into polymer-based NPs designed to cross the BBB or be administered intrathecally [9,12]. In animal models of Alzheimer's, this approach led to enhanced BDNF expression in the brain and improvements in synaptic plasticity and cognitive function, suggesting it countered some disease-related neurodegeneration [95]. Similarly, for stroke, where a transient boost of neuroprotective factors can salvage vulnerable neurons, mRNA has shown promise. A notable example employed an intraventricular injection of BDNF mRNA (carried by a polyplex nanomicelle) in a rat model of ischemic stroke [96]. Notably, when this therapy was administered 48 h post-ischemia, it significantly improved neuronal survival in the hippocampus and led to better recovery in cognitive tests compared to controls. The mRNA was mainly taken up by astrocytes, which then secreted BDNF and created a neuroprotective environment for neurons. The success of this delayed treatment approach (dosing 48 h after the ischemic event) is clinically significant, as it models a feasible therapeutic window for intervention to mitigate secondary neuronal death following ischemic injury [97]. Of course, brain delivery of mRNA in humans still faces many hurdles. Intrathecal administration of LNPs encoding mRNA is being investigated (drawing on the success of intrathecal antisense oligonucleotides for diseases like spinal atrophy), and further work is needed to ensure safety. Nonetheless, the potential rewards are high: mRNA could be used to express enzymes to correct metabolic disorders in the CNS, to produce neurotransmitters or their synthetic enzymes for conditions like Parkinson's, or to secrete antibodies that target pathological proteins. While each of these applications has been conceptualized, their clinical feasibility requires further investigation. While preclinical work has now identified BBB-crossing LNPs for mRNA (e.g., ligand-guided formulations that transcytose brain endothelium) [9], **the** first human evidence for mRNA in neuro-oncology is more likely to come from immune-mediated approaches, that is, mRNA cancer vaccines that prime systemic T-cell responses against glioblastoma antigens rather than relying on NPs-mediated BBB transport. For example, CureVac's CVGBM is a multiepitope mRNA vaccine in Phase 1 after resection, and BioNTech's oncology programs (e.g., BNT211 CAR-T ± RNA-LPX) illustrate how mRNA can augment anti-tumor immunity; neither constitutes systemic NP-mediated delivery into the brain parenchyma [98].

**Table 3:** Organ-specific targeting strategies.

Target Organ	Key Delivery Barriers	Targeting Strategies
**Lung**	Mucus layer; rapid clearance	Inhalation aerosol delivery for localized high-dose exposure; optimize nanoparticle size for deep lung deposition
**Brain**	Blood–Brain Barrier (BBB)	Ligand-targeted nanoparticles or receptor-mediated transcytosis to cross BBB; direct intrathecal injection for certain cases
**Heart**	Off-target uptake (liver, etc.); need to reach myocardium	Direct myocardial injection during surgery (e.g. intracardiac delivery); design carriers that avoid liver and preferentially release in cardiac tissue
**Tumors**	Dense stroma; high interstitial pressure; heterogeneous perfusion	Exploit Enhanced Permeability & Retention (EPR) effect with small (∼100 nm) LNPs; **+** use intratumoral injections or tumor-targeted ligands to ensure mRNA uptake in tumor cells
**Pancreas**	Digestive enzymes; liver uptake competes with delivery to pancreas	Intraperitoneal administration (shown to improve pancreatic mRNA delivery in rodents); develop organ-tropic lipids or polymers that favor pancreatic accumulation
**Lymph Nodes**	Requires transit into lymphatic system; uptake by immune cells needed	Subcutaneous (intradermal) injection to ferry nanoparticles to lymph nodes; decorate nanoparticles with antibodies or peptides targeting dendritic cells and lymphocytes for direct LN delivery

### 4.3 Pancreas

The pancreas, particularly the insulin-producing islets of Langerhans, is a key target for mRNA therapies aimed at diabetes. In type 1 diabetes (T1D), autoimmune destruction of pancreatic β-cells leads to insulin deficiency. In type 2 diabetes (T2D), β-cells are present but often insufficient or dysfunctional in the face of insulin resistance. Traditional insulin therapy involves regular subcutaneous injections of recombinant insulin protein. An mRNA approach to diabetes could potentially provide more physiological and long-lasting glycemic control by enabling the patient's own cells to produce insulin in a controlled manner. However, delivering mRNA to the pancreas is quite challenging. The organ is located in the abdominal cavity and is not easily accessible for local injections. Systemic delivery of LNPs mostly targets the liver, as discussed, with very little naturally going to the pancreas. Recently, researchers have made progress in organ-selective LNPs for example, formulating NPs with certain endogenous ligands that redirect them to the pancreas. A 2025 study reported that adding a cholecalciferol (vitamin D_3_) derived lipid as a fifth component created ENDO LNPs with ∼99 % pancreas selectivity after IV dosing in mice, likely via VDR-mediated interactions [14]. Complementary routes such as IP or GI microneedle gastric delivery also increase pancreatic exposure in rodents, suggesting multiple translational levers [99,100]. Human translation requires validation due to differences in pancreatic vasculature and receptor expression [101]. This kind of targeting key development could pave the way for mRNA therapies to directly transfect pancreatic cells. One therapeutic concept is to deliver mRNA that can reprogram other pancreatic cells into insulin-producing cells. In T1D, where β-cells are destroyed, researchers have explored converting α-cells (which normally produce glucagon) into surrogate β-cells [102]. mRNA could deliver instructions for key transcription factors or proteins that drive this cell identity switch, effectively replenishing insulin-producing cell mass. Though preliminary, these findings underscore mRNA's utility for transient cellular reprogramming. Another approach is to use mRNA for immune modulation in T1D. Since T1D is an autoimmune disease, an mRNA vaccine could be designed to induce tolerance to islet antigens. In a recent proof-of-concept, scientists created an mRNA vaccine encoding fragments of the pancreatic enzyme GAD65 fused to an immune-tolerizing factor. In diabetic-prone mice, intramuscular injection of this mRNA-LNP vaccine generated regulatory immune responses that slowed the development of diabetes: treated mice had a lower incidence of diabetes and better glucose control than controls [102]. The mRNA vaccine worked by reducing autoreactive T cells and inducing tolerance-promoting cytokines like IL-10. These findings suggest that mRNA can be used not only to replace missing proteins but also to fine-tune the immune system in autoimmune disorders. For type 2 diabetes and metabolic syndrome, mRNA therapies might aim to provide metabolic hormones that are beneficial. One candidate is GLP-1, an incretin hormone that stimulates insulin secretion and has become a major drug target (with several injectable GLP-1 analog drugs on the market). Instead of frequent injections of GLP-1 protein, an mRNA therapy might allow the patient's muscle or liver cells to produce GLP-1 continuously for a period of time. This could act like an internal GLP-1 pump, potentially with fewer injections (perhaps an mRNA injection weekly or monthly). Preclinical studies suggest this approach is feasible [103,104], but clinical translation requires validation. For instance, mRNA encoding the hormone FGF21, which improves metabolic parameters in obesity and type 2 diabetes, has been successfully delivered in animal models, leading to reduced body weight and improved insulin sensitivity over weeks of treatment. In one study, obese mice treated with an mRNA for FGF21 showed reversal of obesity-related metabolic derangements, indicating that mRNA delivery of endocrine factors is feasible and can have potent effects [105]. A key challenge for chronic metabolic therapy will be ensuring safety and avoiding unintended effects in off-target tissues. Pancreas-targeted LNPs like the vitamin-D formulation mentioned above could be highly valuable in this regard by concentrating expression in the pancreas; they might, for example, allow β-cells to be selectively enriched with an mRNA for insulin or for a β-cell proliferative factor, without affecting other organs. Indeed, in a transgenic mouse model, that vitamin-D LNP enabled gene editing specifically in pancreatic β-cells, hinting at the ability to confine genetic treatments to the islets. In summary, mRNA therapeutics hold considerable promise for diabetes and pancreatic disorders. Whether by replacing the missing insulin, modulating the auto-immune response, or reprogramming cell fate, mRNA offers a versatile toolkit. The main hurdle has been delivery to the pancreas, but new targeting strategies are rapidly emerging. Preclinical studies suggest this approach is feasible, though clinical translation requires further validation. Advancements in pancreas-targeted delivery could enable therapies that restore endogenous glycemic control, representing a potential approach shift from the current reliance on exogenous insulin and other medications.

### 4.4 Heart

The heart was one of the first organs where mRNA therapy demonstrated significant promise in preclinical studies. The concept of using mRNA to stimulate cardiac repair after myocardial infarction (MI) has been explored for over a decade, and recent clinical trials are beginning to translate this approach to patients. When the heart muscle is damaged, promoting blood vessel growth and cardiomyocyte survival in the injured area can preserve cardiac function. A leading therapeutic candidate in this context has been vascular endothelial growth factor-A (VEGF-A), a potent angiogenic factor that can help grow new blood vessels to supply the injured heart tissue [87]. Direct delivery of VEGF protein or DNA had shown mixed results previously, but mRNA offers some unique advantages: it provides robust but transient production of VEGF, which may avoid some drawbacks of continuous overexpression. In seminal preclinical experiments in mice and pigs around 2013–2018, researchers injected modRNA encoding VEGF-A into the border zone of infarcted hearts and observed improved recovery of heart function compared to controls [106]. These studies paved the way for a clinical trial of an mRNA therapy called AZD8601 (developed by Moderna and AstraZeneca). AZD8601 is a formulated modRNA encoding VEGF-A, intended for local administration to the heart [91]. In a Phase 2a randomized trial for CABG patients (called EPICCURE), surgeons injected the VEGF mRNA into the myocardium of patients undergoing coronary artery bypass surgery for heart failure [86]. The trial reported that the mRNA was well tolerated, without significant adverse events related to the treatment [107]. Although the study was small, there were signs of potential benefit in patients who received the mRNA had certain trends toward improved cardiac function compared to placebo, though the trial was not designed for definitive efficacy conclusions [108]. These results support the further development of mRNA enhanced cardiac surgeries, though larger trials are required to confirm efficacy. This represented the first clinical application of an mRNA therapeutic in the heart, and importantly it demonstrated safety: injecting naked mRNA (in a buffered solution) directly into human hearts caused no serious arrhythmia, no elevated immune reactions, and no organ toxicity over months of follow-up. The use of “naked” mRNA (without a lipid NPs) in this trial is notable. Since the injections were done directly into the heart muscle, the investigators opted for a simple formulation to avoid any potential complications from NPs in the coronary circulation. This success facilitates expanded investigation of mRNA-based cardiac interventions. Another target being investigated is HGF-much like in the lung and other tissues, HGF in the heart can have regenerative effects, promote angiogenesis and protect cardiomyocytes from apoptosis after ischemic injury. Animal studies have indicated that delivering HGF (by gene therapy or protein) shortly after an MI can reduce scar size and improve cardiac output. An mRNA encoding HGF could therefore be beneficial, potentially providing a synergistic effect by concurrently promoting angiogenesis and conferring cardioprotection. Beyond growth factors, mRNA is being explored for cellular reprogramming and immunomodulation in cardiac therapy. A newly developed approach utilizes mRNA for the *in vivo* generation of CAR T cells, programming them to attack and remove activated cardiac fibroblasts that cause fibrosis after an MI [109]. In a 2022 study, researchers injected an LNP carrying CAR mRNA intravenously in a mouse MI model; the LNP was formulated to transfect T cells in the spleen, converting them into “anti-fibrosis” CAR T cells that then homed to the heart and reduced cardiac fibrosis, leading to improved heart function [110]. This approach, while still early-stage, indicates mRNA's versatility in redirecting immune cells to modulate post-MI remodeling. In terms of delivery, the heart can be targeted by both local and systemic means. For localized effects (e.g., VEGF mRNA), direct intramyocardial injection during surgery or *via* catheter can ensure the mRNA is delivered to the area of interest. For more global modulation (e.g., an anti-fibrosis CAR or an arrhythmia-modulating peptide), intravenous delivery with a targeted LNP might be preferred. One challenge for systemic delivery is that the healthy heart is not a highly permeable organ; it lacks the fenestrations of the liver and has relatively low NP uptake. However, in disease states, increased vascular permeability and inflammation might allow more NPs to accumulate in the heart, which could be leveraged for targeted delivery during a window of opportunity post-infarction [111]. Looking forward, mRNA therapies could be applied to other cardiac conditions as well: for instance, to induce controlled regeneration by expressing developmental factors (a controversial but intriguing idea where mRNA for factors like cyclins or fetal genes are delivered to try to coax adult cardiomyocytes to proliferate). While true heart regeneration in adults remains out of reach, mRNA might at least improve repair processes. In summary, the heart has emerged as a promising target for mRNA therapeutics aimed at regenerating blood vessels, preserving muscle viability, and modulating post-injury remodeling. The early clinical trials like AZD8601 show that the approach is safe and can be integrated into existing cardiac care (see Table 3).

### 4.5 Tumors

Cancer represents a multifaceted therapeutic challenge for mRNA-based approaches. Unlike monogenic disorders, tumors involve aberrant cells that must be eliminated or reprogrammed. mRNA therapeutics in oncology have mainly focused on immunotherapy, harnessing the immune system to attack cancer, as well as on directly arming cells with cytotoxic or modulatory proteins. A prominent application of this technology is in the development of mRNA cancer vaccines. These vaccines use mRNA to encode tumor antigens, thereby instructing the patient's own immune cells to recognize and kill tumor cells bearing those antigens. mRNA vaccines can be personalized: for example, in melanoma, it is feasible to sequence a patient's tumor, identify unique mutated peptide sequences, and then produce an mRNA vaccine encoding a cocktail of those neoantigen peptides. Early trials of personalized mRNA neoantigen vaccines have shown encouraging outcomes. In one recent Phase 2 study in patients with high-risk melanoma, an individualized mRNA vaccine (mRNA-4157, encoding up to 34 patient-specific neoantigens) was given in combination with the checkpoint inhibitor pembrolizumab after tumor resection [112]. The combination significantly improved recurrence-free survival compared to pembrolizumab alone, nearly halving the risk of relapse. This trial, reported in 2023, validates the concept that mRNA vaccines can bolster the immune response against cancer and improve clinical outcomes. Another example is an off-the-shelf mRNA vaccine (BioNTech's BNT111) encoding fixed melanoma-associated antigens; in an early-phase trial, this vaccine plus a PD-1 inhibitor led to durable responses in some patients with refractory melanoma [113]. These vaccines are typically delivered intramuscularly or intradermally, where the mRNA is taken up by antigen-presenting cells that then travel to lymph nodes to activate T cells. The successes in melanoma are now spurring trials in other cancers using both personalized and shared-antigen mRNA vaccines. Besides vaccines, mRNA is being used to encode immunotherapeutic agents that can be produced directly in the patient's body. A key advantage is the ability to encode combinations of proteins in a single formulation. For instance, a newly developed approach in solid tumors is to inject mRNA encoding immune-stimulatory cytokines or co-stimulatory ligands directly into the tumor. This strategy aims to remodel the tumor microenvironment from an immunologically "cold" state to a "hot," pro-inflammatory state, thereby rendering it susceptible to immune-mediated clearance [113]. Moderna has tested an intratumoral mRNA mixture (mRNA-2752) [113] that encodes two cytokines (IL-23 and IL-36γ) plus the T-cell costimulatory ligand OX40L. In a Phase 1 trial in patients with advanced solid tumors, intratumoral injections of this mRNA cocktail, in combination with a checkpoint inhibitor, resulted in local cytokine production and some objective tumor responses even in distant lesions, indicating an abscopal immune effect. Similarly, BioNTech is testing an mRNA encoding IL-12 injected into solid tumors. Early results show that mRNA can be safely delivered into tumors with expression of cytokines, leading to immune cell infiltration of the tumor and occasional tumor regressions. An advantage of the mRNA approach here is that the effect is mainly local (the mRNA remains mostly in the injected tumor site and expresses cytokine there, causing high local concentrations that activate immune cells in the tumor without causing the systemic toxicity that would occur if the cytokine protein were given IV) [113]. Beyond cytokines, mRNA can encode other therapeutic modalities such as bispecific antibodies (which can direct immune cells to tumors), or pro-apoptotic proteins that trigger cancer cell death. In fact, one can envision a multi-pronged attack where an mRNA vaccine primes T cells against tumor antigens and an intratumoral mRNA immunotherapy overcomes the immunosuppressive tumor microenvironment all produced from transient mRNA bursts that naturally decay, potentially offering a safety advantage over gene therapy vectors that persist. Efficient delivery in oncology presents distinct challenges: many tumors have abnormal vasculature and high interstitial pressure, which can hinder the penetration of NPs. However, direct intratumoral injection bypasses this for accessible lesions. For metastatic disease, systemic delivery of mRNA to every tumor site is more difficult, but NP engineers are exploring targeting ligands that home to tumors. Another intriguing cancer application of mRNA is *in situ* CAR T cell generation. CAR T cell therapy normally requires extracting a patient's T cells and genetically modifying them *ex vivo*. An mRNA approach, however, could modify T cells inside the body. We discussed an example in the cardiac context; a similar strategy could be used to generate CAR T cells against cancer. In fact, trials are ongoing where patients receive injections of mRNA coding for a CAR [114,115], formulated to primarily transfect T cells, thus temporarily arming their T cells to attack cancer without the need for cell manufacturing. While still early, this could dramatically simplify cell therapy if effective. Finally, it is worth noting that mRNA-based protein replacement can also play a role in cancer treatment. Some tumors are driven by loss of tumor-suppressor proteins; delivering mRNA for a tumor-suppressor gene specifically to tumor cells could, in theory, reprogram them towards normal behavior or sensitize them to other treatments. For example, delivering mRNA for p53 into p53-deficient tumors might induce cell cycle arrest or apoptosis in the cancer cells. This emerging strategy requires further validation delivering mRNA directly to tumor cells remains challenging, but if tumor-specific delivery improves, this approach could complement other therapies. In summary, the application of mRNA in oncology is multifaceted and highly dynamic. Cancer was actually one of the first areas where mRNA vaccines were trialed (even before COVID), and it continues to be at the cutting edge of mRNA therapeutic development. By encoding cytokines, antigens, antibodies, or receptors, mRNA can orchestrate a potent immune-mediated attack on cancer. The clinical evidence so far from melanoma vaccines to intratumoral therapies-indicates significant potential, with mRNA-based immunotherapies achieving outcomes not previously achieved with conventional monotherapies [116,117]. As delivery technologies and our understanding of tumor immunology improve, mRNA will likely become an integral component of combination therapies for cancer, positioning mRNA-based immunotherapies as potentially a critical component in treatment regimens for refractory cancers.

## 5 Strategies to enhance extrahepatic targeting specificity

### 5.1 Ligand-receptor targeting

**Fig. 6:**
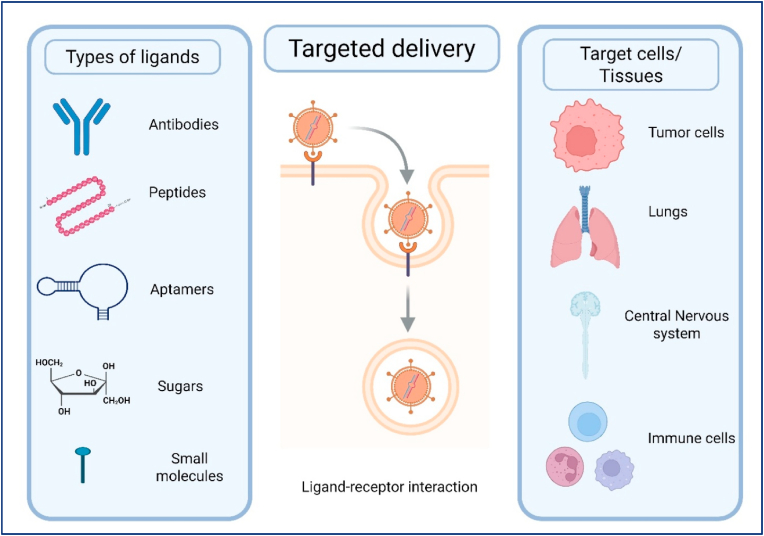
**Various ligands employed to achieve cell-specific delivery of mRNA beyond the liver using receptor–ligand interactions**. Ligands include **antibodies,** which offer high specificity and affinity toward target antigens; **peptides**, which can mimic natural ligands or target overexpressed receptors; **aptamers**, nucleic acid-based molecules with strong and selective binding capabilities; **sugars**, which exploit glycan–receptor interactions for tissue-specific delivery; and **small molecules**, which enable selective targeting through well-defined receptor binding.

Ligand-receptor targeting involves the specific binding of ligands such as hormones, neurotransmitters, cytokines, or synthetic molecules to their corresponding receptors on the cell surface or within the cell (see Fig. 6) hence leads to intracellular signalling cascades [118]. The specificity of ligand-receptor interactions is critical for the fidelity of cellular signalling [119]. For example, G protein-coupled receptors, which represent one of the largest families of cell surface receptors, mediate responses to a diverse array of ligands ranging from small molecules to peptides. They are key targets for approximately 40 % of marketed drugs [120,121]. Monoclonal antibodies have revolutionized cancer treatment for instance, trastuzumab targets the human epidermal growth factor receptor 2 and is overexpressed in certain breast cancers, thereby inhibiting tumor growth and improving patient outcomes [122]. Furthermore, ligand-receptor targeting underpins targeted drug delivery strategies where therapeutic agents are conjugated to ligands recognizing receptors selectively expressed on diseased cells. This approach enhances drug accumulation at the site of pathology and minimizes systemic toxicity [123]. For example, folate receptor-targeted delivery systems exploit the overexpression of folate receptors on cancer cells to improve chemotherapeutic efficacy [124]. Beyond passive chemistry-driven tropism (e.g., SORT; **see** Table 5**)**, ligand functionalization can bias cell entry and organ access.

**Brain (BBB, IV):** peptides that trigger receptor-mediated transcytosis, e.g., Angiopep-2 engaging LRP-1-have repeatedly increased NP exposure in rodent brain and *ex vivo* human tissue slices. [[125], [126], [127]]. **Lung endothelium (IV):** targeting inflamed vascular receptors such as ICAM-1/VCAM-1 yields highly specific lung uptake in rodents and large animals; imaging/PET studies with anti-ICAM carriers show strong pulmonary enrichment. [128,129]. **Solid tumors (IV or intratumoral):** integrin-binding motifs (RGD) and iRGD first bind αv integrins, then, after proteolysis, expose a CendR motif that engages neuropilin-1 to trigger bulk transcytosis and deep intratumoral penetration; co-administration improves delivery of diverse cargos. [130,131]. **Immune cells/lymph nodes (IM/SC/IV):** mannose ligands bias uptake to dendritic cells and macrophages via CD206, improving LN expression and T-cell priming for mRNA vaccines; new DC-targeted LNPs combine stealth and mannose ligands to enhance LN delivery. [132,133]. **Ischemic myocardium (IV/local):** homing peptides identified by *in vivo* phage display (e.g., CSTSMLKAC) enrich particles in infarct zones and can be displayed on polymeric, lipid, or EV-based carriers. [134,135]. **Antibody guidance:** rather than permanently grafting ligands onto LNPs (which can alter size/stealth), bispecific antibody “tethers” that bridge native LNPs to cell markers offer modular, manufacturing-friendly targeting; early reports show cell-specific mRNA delivery *in vivo*. [136,137]. **Caveats:** pooled *in vivo* screens show that ligand benefit depends on corona composition, shear, and dose; benchmarking against the unconjugated parent LNP is essential. **Take-home:** choose ligands that exploit (i) transport pathways absent in the liver, (ii) activated endothelium in diseased tissues, or (iii) pair with route engineering (e.g., inhalation, intratumoral). **See** Fig. 6 for a summary and Table 5 for representative ligand carrier pairs.

Despite its successes, challenges remain, including receptor heterogeneity and adaptive resistance mechanisms. Advances in molecular biology and computational modeling continue to improve ligand design and receptor targeting specificity, promising the development of next-generation therapeutics with enhanced precision and efficacy.

### 5.2 Aptamer-guided delivery

Aptamers and their development of aptamers typically involve the systematic evolution of ligands by exponential enrichment (SELEX), which yields aptamers with optimal binding characteristics to a target molecule [[138], [139], [140]]. Aptamer-guided delivery systems have been widely investigated for cancer therapy; for example, nucleolin-targeting aptamer AS1411 has been conjugated to NPs carrying chemotherapeutic agents to improve uptake in nucleolin-overexpressing cancer cells [141]. Similarly, aptamers targeting prostate-specific membrane antigen have been utilized to deliver siRNA selectively to prostate cancer cells [142]. Aptamer-functionalized nanocarriers can deliver therapeutic nucleic acids such as siRNA or microRNA with high specificity, additionally, aptamers conjugated with imaging agents enable precise molecular imaging [[143], [144], [145]]. Despite promising results, challenges such as nuclease degradation and rapid renal clearance have limited clinical translation. Chemical modifications like 2′-fluoro or 2′-O-methyl substitutions and conjugation with PEG are employed to enhance aptamer stability and circulation time.

### 5.3 Tissue-specific mRNA sequence engineering

Tissue-specific mRNA sequence engineering aims to enhance the precision and efficacy of mRNA-based therapeutics and involves the modification of mRNA sequences such as untranslated regions (UTRs), codon usage, and regulatory elements to achieve controlled spatial and temporal protein expression, thereby minimizing off-target effects [146]. One of the key aspects of tissue-specific mRNA engineering is the optimization of 5′ and 3′ UTRs which play critical roles in mRNA stability, localization, and translational efficiency. By incorporating tissue-specific regulatory motifs or miRNA binding sites into these regions, researchers can restrict mRNA translation [147]. For example, embedding target sites for liver-specific miRNAs in the mRNA can suppress off-target expression in hepatocytes, ensuring more selective delivery to other tissues [148]. Codon optimization tailored to tissue-specific tRNA abundance is another strategy to improve translational efficiency and protein yield in targeted tissues. Codon usage bias varies between tissues, and adjusting codons in the mRNA sequence to match the tRNA pool of the target tissue enhances translation speed and fidelity [[149], [150], [151]]. The clinical relevance of tissue-specific mRNA engineering; for instance, LNP encapsulated mRNAs, as used in COVID-19 vaccines, have prompted further research into mRNA modifications to achieve tissue-specific targeting beyond the liver [152]. Despite these advances, challenges such as innate immune recognition, mRNA degradation, and efficient delivery to non-hepatic tissues remain. Ongoing efforts in chemical modifications of nucleosides, improved UTR designs, and innovative delivery systems aim to overcome these barriers [153].

### 5.4 Emerging concepts in magnetic or ultrasound-guided targeting

Magnetic and ultrasound-guided targeting are innovative, non-invasive techniques designed to enhance the precision of drug delivery and molecular imaging by directing therapeutic agents to specific tissues or pathological sites using external physical forces. These methods represent promising advances in overcoming the limitations of conventional drug delivery systems, such as poor targeting efficiency and systemic toxicity.

#### 5.4.1 Magnetic-guided targeting

Magnetic targeting leverages magnetic fields to guide and concentrate magnetically responsive drug carriers, such as magnetic NPs (MNPs), to desired locations within the body. Magnetic targeting utilizes superparamagnetic NPs, generally consisting of iron oxide (Fe_3_O_4_) or magnetite cores, which react to externally supplied magnetic fields. when the occurrence of a magnetic field gradient around a specified region, such as a tumor, the NPs are subjected to a magnetic force that facilitates their directed movement and aggregation at the location. Upon localization, the field can be sustained to preserve NPs within the tumor microenvironment, mitigating washout due to blood flow. At the cellular level, these magnetically responsive particles can augment uptake via localized hyperthermia or magneto-mechanical stimulation, hence enhancing endosomal escape and intracellular delivery of therapeutic agents such as mRNA [154]. Superparamagnetic iron oxide NPs (SPIONs) are among the most widely studied carriers due to their biocompatibility and strong magnetic responsiveness [155]. Recent advances include the development of multifunctional magnetic nanocarriers capable of simultaneous imaging and therapy, known as “theranostics.” These platforms enable MRI-guided drug delivery, allowing real-time monitoring of NPs distribution and accumulation [156]. Furthermore, magnetic hyperthermia, which involves using alternating magnetic fields to generate localized heat from MNPs, can be combined with chemotherapy for synergistic cancer treatment [157]. Miao and his co-workers et al., Fe_3_O_4_@SiO_2_@Au core–shell NPs (FSA-NPs) have been engineered as multifunctional magnetic theranostic platforms. The Fe_3_O_4_ core facilitates magnetic-field-guided targeting, the mesoporous SiO_2_ shell offers substantial drug-loading capacity, and the gold NPs surface enables photothermal conversion. FSA-NPs, characterized by a particle size of 60–80 nm and a slightly negative surface charge, exhibited effective internalization by A549 lung cancer cells and increased cytotoxicity in the presence of an external magnetic field, underscoring their potential for integrated magnetic targeting, drug delivery, and photothermal therapy in oncological applications [158].

Challenges remain in achieving effective magnetic targeting in deep tissues, to address these issues, researchers are exploring magnetic field gradients, improved NPs surface coatings, and magnetizable implants as local anchors [159].

#### 5.4.2 Ultrasound-guided targeting

Ultrasound guided methods have emerged as a promising approach for enhancing the targeted delivery of RNA interference (RNAi), for instance, Chen et al. (2015) demonstrated that ultrasound-guided injection of lentiviral vectors encoding siRNA towards hypoxia-inducible factor-1 alpha (HIF-1α) resulted in effective gene silencing within orthotopic liver tumors in rats. The combination of siRNA transfection and transarterial chemoembolization (TACE) significantly promoted survival, reduced tumor volume, decreased angiogenesis, and suppressed metastasis compared to either treatment alone [160]. Ultrasound can increase tissue permeability and promote localized drug release through mechanisms such as cavitation, acoustic streaming, and thermal effects [161]. Microbubbles gas-filled lipid or polymer spheres are commonly employed as ultrasound contrast agents and drug carriers. When exposed to ultrasound, microbubbles oscillate and can transiently disrupt cell membranes or the blood-brain barrier, facilitating targeted drug delivery [162]. Ultrasound-generated microbubbles in the lungs can mechanically reduce or split the mucus layer, so improving NPs dispersion and epithelial uptake, while also causing transient cell membrane permeabilization (sonoporation) that aids in cytosolic mRNA release. Tsuruta et al. developed a cationic microbubble formulation that exhibited significant electrostatic adherence to respiratory mucus, hence increasing picture contrast post-inhalation without epithelial absorption or toxicity. The drug exhibited no proinflammatory or cytotoxic effects on human bronchial epithelial cells *in vitro*, while *in vivo* experiments in mice demonstrated superior tolerability and improved tracheal visibility relative to saline controls. The findings indicate mucus-targeting, ultrasound-responsive microbubbles can safely assimilate with the mucociliary layer and enhance pulmonary ultrasound imaging, potentially providing a noninvasive alternative to existing ionizing diagnostic techniques for airway diseases [163]. Clinical translation of ultrasound-guided delivery systems is ongoing, with several trials demonstrating safety and preliminary efficacy in cancer and neurological diseases [164]. However, challenges such as optimizing ultrasound parameters for tissue-specific effects, controlling off-target bioeffects, and ensuring reproducibility remain [165].

#### 5.4.3 Future perspectives

Integrating magnetic and ultrasound-guided targeting with other modalities, such as ligand-receptor recognition or aptamer targeting, is an emerging concept aimed at further enhancing targeting specificity and therapeutic outcomes.

## 6 Manufacturability of novel carriers for mRNA delivery

Manufacturing mRNA delivery carriers, especially LNPs, demands scalable and reproducible processes to ensure batch-to-batch consistency. Microfluidic mixing techniques have become the gold standard for LNP production, enabling rapid and controlled NPs assembly through the mixing of an ethanol phase containing lipids with an aqueous phase containing mRNA [166]. Additionally, microfluidic platforms employed for RNA and its synthesis require materials that maintain structural and functional integrity during prolonged continuous operation. Hwang et al. (2025) comprehensively demonstrated the durability of polymeric materials used in microfluidic mixing units, highlighting the susceptibility of conventional materials such as PDMS to swelling, cracking, and surface degradation. The study emphasized the integration of chemically resistant materials like cyclic olefin copolymers (COCs) and fluoropolymers, which exhibit superior solvent resistance and mechanical stability under continuous flow. These advancements inform the design of robust microfluidic reactors essential for scalable, reproducible RNA LNPs production [167]. The transition from laboratory-scale microfluidic devices to industrial-scale continuous flow reactors remains an ongoing challenge and efforts to optimize flow rates, mixing geometries, and lipid formulations are essential [168]. Novel carriers for mRNA delivery primarily use ionizable lipids, PEG-lipids, cholesterol, and helper phospholipids. The selection of lipids with favourable biodegradability and low toxicity profiles is paramount to meet regulatory requirements and minimize adverse effects [169]. Additionally, raw material supply chain robustness and purity are critical to ensure reproducibility and regulatory compliance [170]. Beyond LNPs, polymeric NPs, lipid-polymer hybrids, and exosome-mimetic vesicles are emerging as alternative carriers [171]. Nonetheless; Robust analytical methods are employed to monitor mRNA integrity, particle size, encapsulation efficiency, and potency over time [137]. Potency assays, including *in vitro* transfection efficiency and expression studies, evaluate functional stability [20]. Stability studies under accelerated and real-time conditions guide shelf-life determination and inform storage recommendations in regulatory submissions (ICH Q1A(R2), 2003). Emerging strategies to enhance formulation stability include the design of novel ionizable lipids with improved biodegradability and membrane fusion properties, and the development of thermostable formulations that remain stable at 2–8 °C or room temperature, significantly easing distribution constraints [172,173]. Additionally, advanced lyophilization protocols combined with excipient optimization hold promise for ambient-temperature stable mRNA therapeutics [169]. The stability and shelf life of mRNA formulations are critical determinants of their efficacy, safety, and commercial viability. Unlike traditional small-molecule drugs, mRNA molecules are inherently labile and susceptible to degradation through hydrolysis, enzymatic activity, and oxidation, necessitating carefully optimized delivery formulations that protect the payload during storage, transport, and administration [169].

Encapsulation of mRNA within LNPs or other nanocarriers offers substantial protection from enzymatic degradation and improves cellular uptake. LNPs shield mRNA from RNases and reduce exposure to environmental stressors [166]. The physicochemical properties of the carrier, such as lipid composition and particle size, also influence formulation stability. For instance, inclusion of cholesterol and PEGylated lipids enhances membrane rigidity and steric stabilization, respectively, thereby improving shelf life [173]. If we come to a different possibility, the stability of mRNA within LNPs can be compromised by the interaction between mRNA and lipid. For instance, Hashiba et al. (2024) demonstrated that aldehyde impurities generated through oxidation and hydrolysis of ionizable lipids can covalently bind to mRNA nucleosides, forming mRNA-lipid adducts that suppress mRNA integrity and translation efficacy during storage. Their study revealed that piperidine-based ionizable lipids significantly limit aldehyde generation because of their unique amine headgroup structures, thereby enhancing mRNA-LNP thermostability, hence minimizes chemical modifications [174]. Current mRNA vaccines require stringent cold chain storage to maintain stability Pfizer-BioNTech's BNT162b2 vaccine initially required storage at −70 °C, while Moderna's mRNA-1273 vaccine is stable at −20 °C, reflecting differences in formulation and lipid composition [172]. These low temperatures minimize hydrolysis and enzymatic degradation but pose logistical challenges and efforts to improve thermostability include optimizing buffer systems, lipid compositions, and incorporating cryoprotectants, which stabilize mRNA and NPs by preventing ice crystal formation and maintaining membrane integrity during freezing and thawing [168]. Emerging strategies to enhance formulation stability include the design of novel ionizable lipids with improved biodegradability and membrane fusion properties, and the development of thermostable formulations that remain stable at 2–8 °C or room temperature, significantly easing distribution constraints [172,173]. Additionally, advanced lyophilization protocols combined with excipient optimization hold promise for ambient-temperature stable mRNA therapeutics, broadening their accessibility worldwide [169].

## 7 Regulatory perspectives on mRNA delivery

RNA therapeutics are classified as biologics by the FDA, with several RNA-LNP therapies such as Patisiran (Onpattro) and mRNA COVID-19 vaccines achieving regulatory approval. Current regulatory frameworks from the FDA, EMA (European Medicines Agency), and the International Council for Harmonisation (ICH) guide quality, safety, and manufacturing control for RNA-LNP systems. Emerging platform designation programs aim to expedite regulatory review by leveraging prior data to accelerate development timelines, reflecting the evolving regulatory landscape for RNA delivery technologies [175]. mRNA therapeutics present distinct regulatory considerations compared to traditional small molecules or biologics:

• **Complexity of Formulations:** mRNA drugs are delivered *via* sophisticated carriers, typically LNPs, which are complex, multi-component systems that must be characterized in detail for size, morphology, composition, and stability [176]. This complexity challenges standard analytical methods and necessitates the development of novel characterization tools [20].
• **Manufacturing and Quality Control:** The production process must ensure reproducibility and purity of both mRNA and carrier components, with strict control of critical quality attributes (CQAs). Given the sensitivity of mRNA to degradation, GMP compliance and cold chain logistics also become regulatory focal points [177].
• **Safety and Immunogenicity:** Ionizable lipids and other excipients may induce unexpected toxicities or immune responses, necessitating comprehensive preclinical toxicology and immunogenicity studies [178]. Regulatory agencies require detailed assessments to evaluate risks of inflammation, complement activation, and off-target effects.

### 7.1 Regulatory guidance and approvals

Several key regulatory guidelines and frameworks have been issued or updated to accommodate mRNA therapeutics:

• The FDA has issued guidance on chemistry, manufacturing, and controls (CMC) considerations for lipid NP drug products, emphasizing characterization of lipid components, particle size distribution, encapsulation efficiency, and stability (FDA, 2020) [179].
• The EMA has provided reflection papers and guidelines on nanomedicine, addressing regulatory expectations for complex formulations, including mRNA-LNPs, and encouraging early dialogue with developers to address unique challenges (EMA, 2018) [180].
• The ICH guidelines, including Q8 (Pharmaceutical Development), Q9 (Quality Risk Management), and Q10 (Pharmaceutical Quality System), are applied flexibly to mRNA products, with agencies recognizing the need for iterative data submission as these novel therapies evolve (ICH, 2020) [181].

### 7.2 Specific examples of regulatory-approved mRNA delivery products

• **Pfizer-BioNTech BNT162b2 (Comirnaty):** This LNP-formulated mRNA vaccine against SARS-CoV-2 was the first mRNA therapeutic granted Emergency Use Authorization (EUA) by the FDA in December 2020 and full approval in August 2021 [181]. Regulatory review emphasized LNP composition, manufacturing controls, mRNA integrity, and extensive safety data.
• **Moderna mRNA-1273 (Spikevax):** Also granted EUA and subsequent approval by FDA and EMA, Moderna's vaccine uses a proprietary ionizable lipid in LNPs. Regulatory agencies closely evaluated the manufacturing process, stability data, and immunogenicity profile of the lipid carrier alongside the mRNA (EMA, 2021) [182].
• **CureVac CVnCoV:** An mRNA vaccine candidate employing a non-chemically modRNA platform, which was under regulatory review. Although not ultimately approved, its clinical evaluation provided regulatory agencies with additional data on mRNA delivery variability and challenges (CureVac AG, 2021) [183].

### 7.3 Adaptive regulatory approaches and future outlook

Regulators have adopted accelerated and adaptive pathways to expedite mRNA therapeutics’ development during the pandemic, including rolling submissions and EUA mechanisms. These experiences are informing long-term regulatory strategies that balance rapid access with rigorous evaluation of novel delivery systems [20].

Going forward, regulators emphasize the importance of:

• Establishing standardized analytical and potency assays for mRNA and carriers
• Enhancing pharmacovigilance for long-term safety monitoring
• Facilitating harmonized global standards to support multinational development and distribution [184].

The regulatory landscape continues to evolve as mRNA therapeutics expand beyond vaccines into areas such as cancer immunotherapy, rare diseases, and gene editing, necessitating ongoing collaboration between industry, academia, and regulatory bodies.

## 8 Clinical and preclinical development landscape

**Fig. 7:**
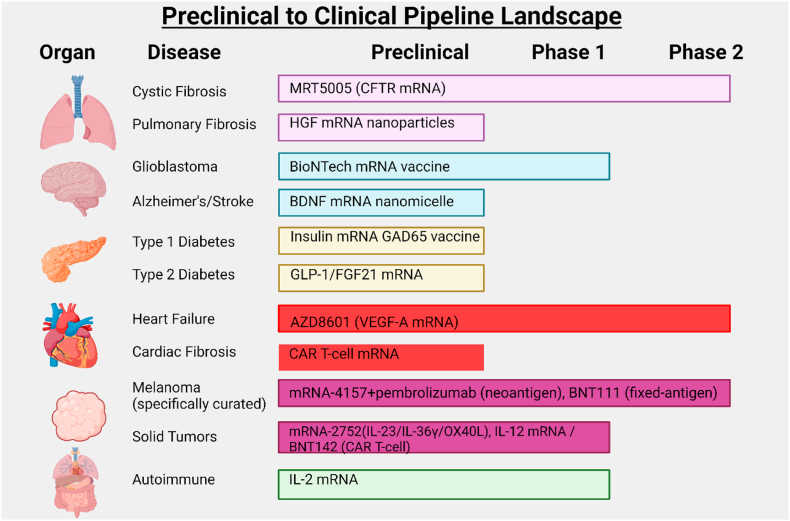
**Preclinical to clinical development stages across organs**. Preclinical-to-clinical pipeline of mRNA therapeutics across extrahepatic organs. The figure maps disease targets and stages of development for lungs, brain, pancreas, heart, tumors, and autoimmune disorders. Graphical representation of Table 3.

mRNA non-vaccine therapeutics are advancing into preclinical and clinical trials, and the extrahepatic delivery of mRNA significantly broadens the therapeutic scope beyond the liver**.** The mRNA Delivery Systems 2.0 focus on engineering LNPs which utilize SORT LNPs to overcome biological hepatic barriers and selectively deliver RNA to extrahepatic tissues such as lungs, spleen, muscle, heart, lung and brain. SORT utilizes auxiliary lipids to redirect tropism, and exosome**-**based carriers leverage natural vesicles for biocompatible delivery (see Fig. 7). The mRNA non-vaccine therapeutics have functional outcomes, including the robust mRNA expression in non-hepatic tissues, wherein lung stem cells achieved over 70 % editing efficiency persisting for >660 days, siRNA SORT LNPs yielded 60–80 % knockdown in lungs and spleen, and ∼15 % in kidneys after single IV doses.

### 8.1 Survey of trials for non-vaccine mRNA therapeutics

The non-vaccine mRNA therapeutics have reached clinical trials for several conditions, including inherited disorders, metabolic, cardiovascular and immunotherapeutic conditions. The candidates, including mRNA-3927 (Propionic acidemia), mRNA-3705 (Methylmalonic acidemia), and mRNA-3745 (Glycogen storage disease type 1a) (see Table 4) utilize IV LNP delivery to replace deficient enzymes, demonstrating promising early efficacy and safety signals [137,[185], [186], [187], [188]]. Preclinical investigation on mRNA-3210 for PKU, mRNA-3351 for Crigler-Najjar, further highlights the scope of this therapeutic model for inherited metabolic disorders [189,190]. mRNA therapies like ARCT-810 for OTC deficiency and Exosome LDLR mRNA for familial hypercholesterolemia employ exosome-based systems aiming to improve targeting and reduce immune reactions [191]. MRT5005 represents an innovative inhaled mRNA therapy for cystic fibrosis, marking progress toward localized respiratory delivery and potential direct organ-targeted treatment. mRNA-1944 for chikungunya infection uses systemic delivery to encode protective antibodies, expanding the role of mRNA beyond vaccines to passive immunotherapy [86]. AZD-8601 uses intracardiac injection of VEGF-A mRNA to stimulate angiogenesis post-coronary artery bypass graft surgery, exploring the potential of mRNA therapeutics in regenerative medicine [86,91] (see Table 4).

**Table 4:** Various investigational mRNA therapeutics and gene therapies in rare metabolic and genetic disorders.

Molecule	Indication	Trial ID	Phase	Route	Key Results/Notes	Reference
mRNA-3927	Propionic acidemia (enzyme replacement)	NCT04159103	Phase 1/2	IV (LNP)	∼70 % reduction in metabolic decompensation events; well tolerated	[197]
mRNA-3705	Methylmalonic acidemia	NCT04899310	Phase 1/2	IV (LNP)	No clinical readout yet; selected for FDA START pilot program	[199]
mRNA-3745	Glycogen storage disease type 1a	NCT05095727	Phase 1	IV (LNP)	Early safety data; systemic enzyme replacement strategy	[198]
ARCT-810	Ornithine transcarbamylase deficiency (OTCD)	NCT06488313	Phase 2	IV (LNP)	Phase 2a ongoing; fast track designation by FDA	[200]
MRT5005	Cystic fibrosis (CFTR gene delivery via inhalation)	NCT03375047	Phase 1/2	Inhalation (LNP)	Early-stage trial; demonstrated safety and biological activity	[232]
mRNA-3210	Phenylketonuria (PKU)	Preclinical	Preclinical	IV (LNP)	Enzyme replacement in preclinical models	[189]
mRNA-3351	Crigler-Najjar syndrome (CN-1)	Preclinical	Preclinical	IV (LNP)	Enzyme replacement in preclinical models	[190]
mRNA-1944	Chikungunya (antibody generation)	NCT03829384	Phase 1	IV (LNP)	Positive safety and activity data; systemic mRNA delivery of antibodies	[191]
Exosome LDLR mRNA	Familial hypercholesterolemia	NCT05043181	Phase 1	IV (exosome-based)	Early safety and PK data	[233]
AZD-8601	VEGF-A mRNA for therapeutic angiogenesis (post-CABG)	NCT03370887	Phase 2a	Intracardiac injection	VEGF expression and angiogenesis post-cardiac surgery	[234]

**Table 5:** Lipid nanoparticle (LNP) libraries for mRNA delivery: composition, payloads, and functional applications.

Library Name/System	Library Size	Payload Type	Key Features/Applications	References
CAD Lipid Library	∼180 diverse lipids	Luciferase mRNA + DNA barcode	Cationic degradable lipids; pooled IV in mice	[170]
FIND (Functional In Vivo Delivery) System	∼137 LNPs	Cre mRNA + DNA barcode	Functional delivery via Cre recombinase; fluorescence reporter	[210]
Oxidized LNP (oLNP) Library	61 LNPs	mCherry mRNA + ssDNA barcode	Oxidized vs unoxidized lipids; immune cell targeting	[34]
SORT LNP Libraries	Variable	mRNA + barcode	Modulation of helper lipids charge (cationic/anionic)	[211]
LipoBART (Sanofi)	∼100+ LNPs	Barcoded mRNA + DNA	Machine learning-driven LNP design	[212]

### 8.2 Emerging players: Orna Therapeutics (circular mRNA), capstan (muscle), arcturus (lung)

The emerging players in the non-vaccine mRNA therapeutics are rapidly advancing innovative platforms that expand the potential of mRNA beyond traditional vaccine applications. Orna Therapeutics is pioneering the use of circular RNA (circRNA), which offers enhanced stability and prolonged protein expression compared to conventional linear mRNA [192]. The molecules in the pipeline including ORN 101 aims to target B cell-driven autoimmune diseases and B cell malignancies, supported by panCAR™ platform [193]. Capstan Therapeutics is leading the muscle-targeted delivery of multi-mRNA cocktails, through a novel approach of the proprietary CellSeeker™ platform, which utilizes tLNPs to deliver multi-mRNA cocktails directly into muscle cells [194]. This approach is still early in development, with preclinical results showing the tLNPs can effectively deliver mRNA encoding an anti-CD19 CAR to CD8^+^ T cells, leading to rapid and profound anti-tumor and anti-primary B cell activity in mice [195]. Arcturus Therapeutics has developed advanced LNP and self-amplifying mRNA technologies enabling efficient delivery to hepatic and pulmonary tissues [196]. Their clinical pipeline includes candidates like ARCT-810 for ornithine transcarbamylase deficiency and an intramuscular COVID-19 vaccine, showcasing versatility across therapeutic areas [197]. The mRNA candidates of Moderna continue to expand its robust pipeline of linear mRNA therapeutics targeting rare metabolic diseases, immuno-oncology, and infectious diseases through various delivery routes, including intravenous, inhalation, and intramuscular [[198], [199], [200]].

### 8.3 Case studies of pipeline candidates in oncology, cardiology, and rare diseases

In propionic acidemia (PA) mouse models, systemic LNP delivery of dual mRNA-3927 encoding both PCC subunits restored hepatic enzyme activity and significantly reduced levels of disease biomarkers, with consistent efficacy over 3–6 months post-dose and robust pharmacokinetics across mice, rats, and nonhuman primates supporting rapid protein expression within 6 h and informing first-in-human dosing [201]. Similar preclinical studies of mRNA-3705 (for MMA) and mRNA-3210 (for PKU) in mouse models demonstrated strong PK/PD responses, with normalization of respective disease biomarkers and validated allometric models for clinical translation [202]. In oncology, intratumoral LNPs delivering mRNA-2416 (OX40L) in murine H22 hepatocellular carcinoma resulted in tumor growth inhibition, amplified CD4^+^/CD8^+^ T-cell infiltration, and 50 % complete response rates, including immune memory upon rechallenge [203]. Other mRNA vaccines encoding cytokine cocktails in mouse models induced potent antigen-specific T-cell responses, delayed tumor growth, and supported development of immunomodulatory intratumoral mRNA therapies [204].

In Phase 1 clinical testing, intratumoral OX40L monotherapy in 39 patients exhibited a favourable safety profile (no dose-limiting toxicities), with 6/14 evaluable patients achieving stable disease for 14 weeks and 4 demonstrating tumor shrinkage both at injected and distant sites, accompanied by increased intratumoral OX40L expression and pro-inflammatory gene signatures [205]. For cancer immunotherapy, the KEYNOTE-942 Phase 2b study of mRNA-4157 plus pembrolizumab showed a 44–49 % reduction in melanoma recurrence risk at 18 months (RFS: 79 % vs 62 %; HR ∼0.56), with sustained 3-year benefits and acceptable safety [87,91,206]. Additionally, Phase 1 trials of mRNA-1944 delivering chikungunya antibodies demonstrated favourable safety and measurable antibody titers, while an mRNA-based rabies vaccine in first-in-human cohorts elicited both strong immunogenicity and tolerability. These extensive studies illustrate the robust capabilities of mRNA therapeutics in animal models and human trials.

### 8.4 Translational insights: what enables successful extrahepatic delivery in humans?

Extrahepatic mRNA delivery in human studies stems from a combination of factors, including NPs engineering and charge on lipids, particle size, target molecules, carrier engineering, polymers, type of RNA, exosomes, and route of administration. LNPs containing ionizable lipids facilitate endosomal escape with minimal systemic toxicity as evidenced by the LUNAR-enabled ARCT-810 maintaining liver specificity in early-phase trials without significant lung or spleen exposure [207]. Meanwhile, charge and particle size tuning shaped the development of inhaled MRT5005, which achieved successful pulmonary delivery in a Phase 1/2 cystic fibrosis trial; it was well tolerated with mild febrile and hypersensitivity reactions, and no consistent improvements in FEV_1_, confirming localized lung exposure without systemic distribution. The concept of targeting ligands and conjugates is supported by ongoing efforts using GalNAc and peptide-guided LNPs in clinical programs, although specific results remain forthcoming [208]. Route of administration also governed the effective extrahepatic mRNA delivery in intratumoral for OX40L and intramuscular for mRNA-4157 (neoantigen vaccine), which has demonstrated localized immune activation and systemic therapeutic effects in human patients. The chemical modifications to mRNA, including the incorporation of N1-methylpseudouridine and the use of circRNA constructs, have enhanced translational efficiency, stability, and safety profiles underpinning progress across both preclinical and clinical settings [209]. These combined advances are facilitating the successful translation of extrahepatic mRNA therapies from murine models to human studies.

## 9 Strategic roadmap and future perspectives

### 9.1 Barcoded LNP libraries and *in vivo* tropism mapping

The use of barcoded LNP libraries provided the first real opportunity to examine mRNA delivery tropism *in vivo* by facilitating high-throughput, quantitative mapping of tissue and cell-specific transfection. LNP libraries are composed of hundreds of chemically diverse LNP formulations each unique(s). Barcoded LNP libraries have optimized the study of mRNA delivery tropism *in vivo* by enabling high-throughput, quantitative mapping of tissue and cell-specific transfection. LNP libraries consist of hundreds of chemically diverse LNP formulations that are each uniquely labelled with a nucleic acid-based barcode that can be sequenced after it is administered to identify biodistribution and functional delivery profile simultaneously. The Cationic Degradable (CAD) lipid library is composed of a diverse set of cationic degradable lipids and the aim of the library is to identify LNPs that efficiently deliver the mRNA cargo (including luciferase) to the lungs in mice [170]. The FIND (Functional *In Vivo* Delivery) system uses 19 different cell types with a combination of Cre-recombinase mRNA (with barcodes) to assess functional delivery across all cell types and uses fluorescence reporters to identify the successful cellular transfection process [170,210]. The oxidized LNP library allows for a direct comparison between oxidized and non-oxidized lipids and allows for LNP formulation screening to identify LNPs that target immune cells, like monocytes, which is important for applications like *in vivo* CAR-monocyte engineering [34]. The SORT lipid formulation is based on adjusting the charge on the helper lipid in order to identify the optimal delivery to a specific target, like endothelial cells of the lung or cells from the spleen or liver. There are also applications like LipoBART that use machine learning and combine the barcoded libraries with the information to determine the optimal design per tissue [211] (see Table 2).

### 9.2 AI/ML in carrier design, biodistribution prediction, and patient stratification

AI and ML are becoming increasingly key to the advancement of mRNA therapeutics by providing host of opportunities to improve carrier design, predict biodistribution patterns, and optimize patient stratification. In relation to carrier design, ML methods are being used to search for chemical libraries of LNPs to find structural properties that improve delivery and tissue specificity. As an example, a random forest regression model was then able to predict characteristics of >80 LNPs tested *in vivo* for lung delivery based on the LNP physicochemical properties and biomarker expression in the lung [212,213]. Computational models have also been built to predict biodistribution patterns on the basis of LNP physicochemical properties that allow for rational design decisions before *in vivo* testing [214,215]. These models generally include parameters such as lipid type, size, charge, and PEGylation, which influence the circulation time and tissue uptake of the NPs. In direct applications, AI-based patient stratification permits mRNA therapeutics to develop personalized approaches by analyzing patient-specific genetic, molecular, and phenotypic data to provide mRNA treatments or delivery platforms to patients who are most likely to benefit [216]. For example, in recent oncology trials, AI was used in tumor mutational burden and immune profile categorization, producing personalized cancer vaccines with mRNA [217,218]. Additionally, advancing predictive modelling by integrating ML with imaging, pharmacokinetic data to realize continuous real-time predictive outcome measures to not only understand outcome measures but also to anticipate adverse effects to improve safety and efficacy. AI/ML tools simplify and streamline the complex optimization of mRNA therapeutics with predictive analytics ranging from the early preclinical screens to clinical use, greatly reducing translational timelines.

### 9.3 CRISPR-mRNA integration and the scope of personalized medicine

Delivering CRISPR-associated proteins, including Cas9, base editors, or prime editors as mRNA allows transient, controllable, and non-integrative editing of disease-causing genes *in vivo*, reducing off-target effects and immunogenicity compared to DNA-based delivery [219]. *In vivo* gene editing *via* LNP-delivered Cas9 mRNA has been used to guide RNAs to correct mutations in mouse models of liver disease [220]. Building upon this, base editors, mRNA-encoded fusion proteins capable of single-nucleotide conversions without double-strand breaks, have shown promise in correcting point mutations with high precision and minimal DNA damage [221]. Prime editing, a more recent innovation, utilizes a reverse transcriptase fused to Cas9 and programmed by a prime editing guide RNA; its mRNA delivery is currently under preclinical investigation, offering versatile correction capabilities for diverse genetic alterations [222]. Recent research has shown that optimizing lipid composition and biomaterial design can target CRISPR–mRNA or RNP to extrahepatic tissues, resulting in quantifiable editing effects. Chen et al. developed thermostable Cas9 RNP–LNPs that attained approximately 37 % editing in the liver and 16–19 % editing in the lung utilizing Ai9 and SFTPC reporter mice [223]. Sun et al. utilized Lung-SORT LNPs to administer adenine base editors, achieving over 70 % editing of lung stem cells and sustained reporter activation in over 80 % of lung epithelial cells. The findings validate that strategic lipid and biomaterial engineering can convert CRISPR–mRNA systems into effective extrahepatic gene-editing platforms; however, comprehensive cross-organ comparisons and standardized efficiency metrics (e.g., percentage of edited cells per organ, NGS-validated indel/correction rates) are necessary to facilitate clinical translation [224].

Parallel to gene editing advances, personalized medicine is rapidly embracing modular, programmable mRNA platforms that enable tailored therapeutic regimens. These systems leverage customizable mRNA sequences encoding patient-specific antigens or therapeutic proteins, combined with flexible delivery vehicles, to address heterogeneous diseases such as cancer or rare genetic disorders [225]. Personalized cancer vaccines using mRNA encoding neoantigens identified from individual tumors have entered clinical trials, demonstrated the feasibility of rapid, bespoke vaccine production, and elicited robust immune responses [226]. The modularity of mRNA allows co-delivery of multiple therapeutic cargos, including immunomodulators or corrective enzymes, facilitating combinational therapies optimized per patient profiles [227]. This programmability is further enhanced by advances in mRNA chemical modifications and delivery technologies, ensuring stability and targeted expression in desired tissues.

### 9.4 Emerging ethical and access challenges

The rapid advancement of mRNA therapeutics, especially in personalized and gene-editing applications, has brought emerging ethical and access challenges that warrant careful consideration. One key ethical concern center around equitable access, as these innovative therapies often come with high development and production costs, potentially exacerbating healthcare disparities between wealthy and low-resource settings [228]. Additionally, the transient yet powerful nature of mRNA-based gene editing, such as CRISPR-mRNA systems, raises questions about long-term safety, unintended off-target effects, and the ethical implications of germline modifications, despite current focus on somatic editing [229]. Patient consent and transparency are further complicated by the highly personalized nature of many mRNA treatments, where rapid customization may limit extensive preclinical safety data and long-term follow-up [230]. Moreover, intellectual property and patent protections on novel mRNA platforms and delivery systems can restrict generic production, influencing global availability and pricing. Ethical frameworks are also evolving to address dual-use risks, such as potential misuse of gene-editing technologies for non-therapeutic enhancements or bioweapons [231].

## 10 Conclusion and opinion

**Fig. 8:**
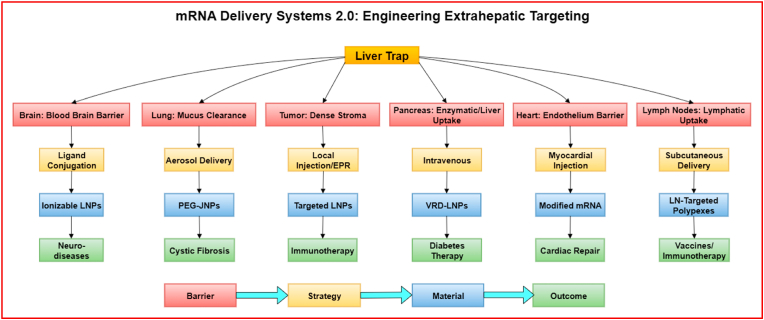
Conclusion flow chart across organs.

The landscape of mRNA therapeutics has evolved rapidly, transitioning from liver-centric (see Fig. 8), vaccine-focused applications to a broader spectrum of extrahepatic, non-vaccine strategies. This progression is underpinned by significant advancements in LNP technology, library-based screening methods, and computationally guided design, enabling tissue-specific delivery and improved therapeutic indices. In particular, barcoded LNP libraries and functional *in vivo* delivery (FIND) systems have accelerated the identification of NP capable of targeting hard-to-reach organs such as the lung, spleen, muscle, and immune cells, offering a translational roadmap toward addressing previously inaccessible disease areas. The clinical and preclinical pipeline now features a growing roster of non-vaccine mRNA candidates targeting rare metabolic disorders, oncology, cardiology, and pulmonary diseases. Emerging companies like Orna Therapeutics, capstan therapeutics, and arcturus therapeutics are pioneering new formats such as circular mRNA, self-amplifying RNA, and platform-specific delivery approaches tailored to muscle or lung tissue. These developments underscore the expanding versatility of mRNA platforms beyond the COVID-19 paradigm. Case studies of lead candidates such as mRNA-based enzyme replacement for PA, VEGF-A mRNA for post-cardiac surgery angiogenesis, and CFTR gene delivery for cystic fibrosis demonstrate proof-of-concept efficacy and safety in early-stage trials, offering hope for patients with limited therapeutic options. A key enabler of this shift toward extrahepatic success lies in the precise tuning of delivery systems. From the modulation of helper lipid charge in SORT LNPs to oxidized lipid libraries targeting immune cells, innovative design strategies are overcoming biological barriers that have historically restricted mRNA delivery to hepatocytes. Furthermore, AI and ML are playing a pivotal role in predicting biodistribution patterns, optimizing particle composition, and even stratifying patients based on predicted responsiveness. The integration of CRISPR systems with mRNA, whether for gene knockout, base editing, or prime editing, further broadens the therapeutic scope, introducing gene editing capabilities without the risks associated with persistent nuclease expression. Looking ahead, mRNA vaccines 2.0 are being tailored for oncology and autoimmune diseases, leveraging antigen discovery platforms, neoantigen prediction algorithms, and personalized delivery approaches. The future of mRNA therapeutics lies in its modularity and programmability, which will enable bespoke treatment solutions and rapid adaptation across indications. However, this horizon also brings ethical and access-related challenges, particularly around equitable distribution, affordability, and the long-term monitoring of gene-modifying therapies. In conclusion, the mRNA field is witnessing a transformative shift toward extrahepatic applications that transcend traditional vaccine development. The convergence of barcoded screening technologies, computational tools, and genome editing capabilities positions mRNA as a cornerstone of next-generation precision medicine. The strategic implications for drug discovery, personalized therapy, and regulatory science are profoundly ushering in a new era where mRNA can be engineered not just to instruct cells, but to therapeutically reprogram them across a multitude of tissues and disease states.

## CRediT authorship contribution statement

**Manoj Dalabehera:** Writing – review & editing, Writing – original draft, Supervision, Investigation, Formal analysis, Conceptualization. **Arnab Ghosh:** Writing – review & editing, Writing – original draft. **Satyajit Mohanty:** Writing – original draft. **Dinesh Kumar Chellappan:** Writing – original draft, Validation. **Shubham Chaudhari:** Resources. **Yogita Ale:** Writing – original draft. **Neelam Poonia:** Software. **Rudra Narayan Subudhi:** Writing – review & editing, Visualization. **Manmeet Kaur Khanna:** Writing – review & editing. **Hae Gyun Lim:** Visualization, Supervision, Investigation.

## Ethical approval

Not Applicable.

## Funding

This work was supported by the 10.13039/501100003725National Research Foundation of Korea (NRF) grants funded by the 10.13039/501100014188Korean government (MSIT) (Nos. 2022R1A5A8023404 and RS-2024-00338853).

## Declaration of competing interest

The authors declare that they have no known competing financial interests or personal relationships that could have appeared to influence the work reported in this paper.

## Data Availability

Data will be made available on request.

## Abbreviation

mRNA: Messenger RNA
LNPs: Lipid nanoparticles
siRNA: Small interfering RNA
PEG: Polyethylene glycol
NPs: Nanoparticles
BBB: Blood brain barrier
TLR: Toll like receptor
RIG-I: Retinoic acid-inducible gene
PLGA: Polylactic coglycolic acid
PBAE: Poly β-Amino ester
CAR: Chimeric antigen receptor
DOTMA: 1,2-di-O-octadecenyl-3-trimethylammonium-propane
DOTAP: 1,2-dioleoyl-3-trimethylammonium-propane
hATTR: hereditary transthyretin
SORT: Selective Organ Targeting
APoE: Apolipoprotein E
LPLR: Low density lipoprotein receptors
RES: Reticuloendothelial system
ABC: Accelerated blood clearance
CARPA: Complement activation-related pseudoallergy
PNMVA: Poly(N-methyl-N-vinylacetamide
PEI: Polyethylenimine
PGA: Poly-glutamic acid
TAM: Tumor-associated macrophages
HTA: Hydroxy tertiary amine
CPP: Cell-penetrating peptides
modRNA: modified mRNA
PNIPAM: Poly N-isopropylacrylamide
CRGD: Cyclic Arg-Gly-Asp
OVA: Ovalbumin
MVBS: Multivesicular bodies
LPP: Lipid polymer particles
LPs: Lipopolyplexes
ROS: Reactive oxygen species
GDNF: Glial cell line-derived neurotrophic factor
BDNF: Brain-derived neurotrophic factors
HGF: Hepatocyte growth factor
SELEX: Systematic evolution of ligands by exponential enrichment
UTR: Untranslated regions
FIND: Functional *in vivo* delivery
